# UniROS: ROS-Based Reinforcement Learning Across Simulated and Real-World Robotics

**DOI:** 10.3390/s25185679

**Published:** 2025-09-11

**Authors:** Jayasekara Kapukotuwa, Brian Lee, Declan Devine, Yuansong Qiao

**Affiliations:** 1 Software Research Institute, Technological University of the Shannon: Midlands Midwest, N37 HD68 Athlone, Ireland; j.kapukotuwa@research.ait.ie (J.K.); brian.lee@tus.ie (B.L.); 2 PRISM Research Institute, Technological University of the Shannon: Midlands Midwest, N37 HD68 Athlone, Ireland; declan.devine@tus.ie

**Keywords:** reinforcement learning (RL), Robot Operating System (ROS), real-time robotics, sim-to-real transfer, concurrent RL environments

## Abstract

**Highlights:**

**What are the main findings?**

- We developed UniROS, a unified ROS-based reinforcement learning framework that supports real-time learning across both simulated and physical robots.
- We demonstrated asynchronous, concurrent control of multiple real and simulated robots using UniROS, effectively reducing latency and improving scalability.

**What are the implications of the main findings?**

- This enables more efficient and realistic training of multiple robotic agents in the real world, addressing the limitations of existing sequential single-robot frameworks.
- It facilitates learning across different robots and network conditions, accelerating the deployment of real-world reinforcement learning systems.

**Abstract:**

Reinforcement Learning (RL) enables robots to learn and improve from data without being explicitly programmed. It is well-suited for tackling complex and diverse robotic tasks, offering adaptive solutions without relying on traditional, hand-designed approaches. However, RL solutions in robotics have often been confined to simulations, with challenges in transferring the learned knowledge or learning directly in the real world due to latency issues, lack of a standardized structure, and complexity of integration with real robot platforms. While the use of Robot Operating System (ROS) provides an advantage in addressing these challenges, existing ROS-based RL frameworks typically support sequential, turn-based agent-environment interactions, which fail to represent the continuous, dynamic nature of real-time robotics or support robust multi-robot integration. This paper addresses this gap by proposing UniROS, a novel ROS-based RL framework explicitly designed for real-time multi-robot/task applications. UniROS introduces a ROS-centric implementation strategy for creating RL environments that support asynchronous and concurrent processing, which is pivotal in reducing the latency between agent-environment interactions. This study validates UniROS through practical robotic scenarios, including direct real-world learning, sim-to-real policy transfer, and concurrent multi-robot/task learning. The proposed framework, including all examples and supporting packages developed in this study, is publicly available on GitHub, inviting wider use and exploration in the field.

## 1. Introduction

Reinforcement Learning in robotics holds promise for providing adaptive robotic behaviors in scenarios where traditional programming methods are challenging. However, applying RL in the real world still faces numerous challenges, including (1) the reality gap [1] between simulation and physical robots, (2) the real-time delay between observation and action, and (3) the difficulty of scaling to multi-robot or multi-task settings. While these challenges often appear independently in the literature, they require a unified solution to make RL practical for robotic systems. A unified solution (framework) to these challenges can be built using the Robot Operating System, as it primarily unifies message types for sensory-motor interfaces, offers microsecond timing functionalities, and is available for most current commercial and research platforms.

Reality Gap: Robot-based reinforcement learning [2,3] usually depends on simulation models for learning robotic applications and transferring the learned knowledge to real-world robots. This stage remains a major bottleneck because most simulation frameworks face challenges in effectively showcasing how to transfer learned behaviors from simulation models to real robots. One of the main challenges is that the currently available robotics simulators cannot fully capture the exact varying dynamics and intrinsic parameters of the real world. Therefore, agents trained in simulation models cannot typically be directly generalized to the real world due to the domain gap (reality gap) introduced by the discrepancies and inaccuracies of the simulators. To overcome this issue, experimenters must perform additional steps to the learning task, which requires incorporating real-world learning [4] and applying Sim-to-real [5] or domain adaptation [6] techniques to transfer the learned policies from simulation to the real world.

Real-time mismatch: Even after addressing these concerns, a key challenge in real-world robotic learning is managing sensorimotor data in the context of real-time scenarios [7]. In robotic RL, ‘real-time’ refers to the ability of the environment to operate at a pace where the robot’s decision-making and execution of actions must occur within a specific time frame. This rapid pace is essential for the robot to interact effectively with its environment, ensuring that the processing of sensory data and the execution of actuator responses are both timely and accurate. This aspect is particularly critical when creating simulation-based learning tasks to transfer learning to real-world robots. Currently, in most simulation-based learning tasks, computations related to environment-agent interactions are typically performed sequentially. Therefore, to comply with the Markov Decision Process (MDP) architecture [8], which assumes no delay between observation and action, most simulation frameworks pause the simulation to construct the observations, rewards, and other computations. In contrast, time advances continuously between agent- and environment-related interactions in the real world. Hence, learning is typically performed with delayed sensorimotor information, potentially impacting the synchronization and effectiveness of the agent’s learning process in real-world settings [9]. Therefore, these turn-based systems do not mirror the continuous and dynamic nature of real-world interactions and can lead to a mismatch in the timing of sensorimotor events compared with real-world situations. These issues stem from the agent receiving outdated information about the state of the environment and the robot not receiving proper actuation commands to execute the task.

Multi-Robot/Task Learning: In modern RL research, there is a growing interest in leveraging knowledge from multiple RL environments instead of training standalone models. One of the advantages of this approach is that it can improve the agent’s learning by generalizing knowledge across different scenarios (domains or tasks) [10]. Furthermore, combining concurrent environments with diverse sampling strategies can effectively accelerate the agent’s learning process [11]. This leveraging process can expose the agent to learning multiple tasks simultaneously rather than learning each task individually (multi-task learning) [12]. This is also similar to meta-learning [13]-based RL applications, where the agent can quickly adapt and acquire new skills in new environments by leveraging prior knowledge and experiences through learning-to-learn approaches. Another advantage of concurrent environments is scalability, which allows for the simultaneous training of multiple robots in parallel, either in a vectorizing fashion or for different tasks or domain learning applications [14]. Therefore, creating concurrent environments is crucial for efficiently utilizing computing resources to accelerate learning in real-world applications, where multiple robots must be trained and deployed efficiently. While several solutions such as SenseAct [9,15] exist in the literature for real-time RL-based robot learning, they predominantly focus on single-robot scenarios or systems comprising robots from the same manufacturer, limiting their applicability in heterogeneous multi-robot settings [16]. Furthermore, they often overlook the computational challenges inherent in scaling to multiple robots, particularly the CPU bottlenecks that can arise from processing data from various sensors, such as vision systems that may require CPU-intensive preprocessing operations [17,18].

Programming Interface Fragmentation: Another challenge is the programming language gap between simulation frameworks and real-world robots from different manufacturers. Most current simulation frameworks used in RL are commonly implemented in languages like Python, C#, or C++. However, real robots typically have proprietary programming languages, such as RAPID, Karel, and URScript, or may utilize the Robot Operating System (ROS) for communication and control. Therefore, it is not possible to transfer the learned knowledge directly without recreating the RL environment in the recommended robot programming language to communicate with the physical hardware [9]. Furthermore, this challenge also applies when learning needs to occur directly in the real world without relying on knowledge transferred from a digital model. These include cases such as dealing with liquids, soft fabrics, or granular materials, where the physical properties are challenging to model precisely in simulations [19]. In these scenarios, the experimenters must establish a communication interface with the physical robots to enable the agent to directly interact with the real world. This process becomes more challenging if the task requires robots from multiple manufacturers, as they typically do not share a common programming language.

Fortunately, the Robot Operating System (ROS) presents a promising solution to some of these challenges. This is because ROS is widely acknowledged as the standard for programming real robots, and it receives massive support from manufacturers and the robotics community. This makes it an ideal platform for constructing learning tasks applicable to simulations and real-world settings. Currently, numerous simulation frameworks are available for creating RL environments using ROS, with most prioritizing simulation over real-world applications. A fundamental limitation of these simulation frameworks, such as OpenAI_ROS (http://wiki.ros.org/openai_ros, accessed on 17 June 2025), gym-gazebo [20], ros-gazebo-gym (https://github.com/rickstaa/ros-gazebo-gym, accessed on 17 June 2025), and FRobs_RL [21], is their inability to support the creation of real-time RL simulation environments due to their use of turn-based learning approaches. Therefore, the full potential of ROS for setting up learning tasks that can easily transfer learning to the real world is not utilized correctly. Furthermore, with the current offerings, ROS lacks Python bindings for some crucial system-level features needed to create RL environments, such as launching multiple ROScores, nodes, and launch files, which are currently confined to manual configurations (Command Line Interface—CLI approaches). Moreover, the full potential of ROS in creating real-time RL environments that achieve precise time synchronization, which is essential for aligning the sequence and timing of sensor data acquisition, decision-making processes, and actuator responses, thereby reducing latency in agent-environment interactions, has not been thoroughly studied yet. Addressing these gaps in ROS could further streamline the development of effective and efficient RL environments for robots.

Therefore, this study addresses the central question of “How to design an ROS-based reinforcement learning framework that supports both simulation and real-world environments, real-time execution, and concurrent training across multiple robots or tasks”. This paper presents a comprehensive framework designed to create RL environments that cater to both simulation and real-world applications. This includes adding support for ROS-based concurrent environment creation, a requirement for multi-robot/task learning techniques, such as multi-task and meta-learning, which enables the simultaneous handling of learning across multiple simulated and/or real RL environments. Furthermore, this study explores how this framework can be utilized to create real-time RL environments by leveraging an ROS-centric environment implementation strategy that bridges the gap between transferring learning from simulation to the real world. This aspect is vital for ensuring reduced latency in agent-environment interactions, which is crucial for the success of real-time applications.

Furthermore, this study introduces benchmark learning tasks to evaluate and demonstrate some use cases of the proposed approach. These learning tasks are built around the ReactorX200 (Rx200) robot by Trossen Robotics and the NED2 robot by Niryo and are used to explain the design choices. This study also lays the groundwork for multi-robot/task learning techniques, allowing for the sampling of experiences from multiple concurrent environments, whether they are simulated, real, or a combination of both.

Summary of Contributions:

- Unified RL Framework: Development of a comprehensive, ROS-based framework (UniROS) for creating reinforcement learning environments that work seamlessly across simulation and real-world settings.
- Concurrent Env Learning Support: Enhancement of the framework to support vectorized [22] multi-robot/task learning techniques, enabling efficient learning across multiple environments by abstracting standard ROS communication tools into a reusable structure tailored for RL.
- Real-Time Capabilities: Introduction of a ROS-centric implementation strategy for real-time RL environments, ensuring reduced latency and synchronized agent-environment interactions.
- Benchmarking and Evaluation: Empirical demonstration through benchmark learning tasks, addressing these challenges using the proposed framework in three distinct scenarios.

## 2. Background

### 2.1. Formulation of Reinforcement Learning Tasks for Robotics

**Figure 1:**
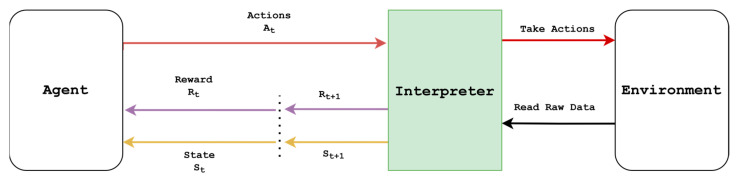
Basic reinforcement learning diagram depicting the critical components, including the agent, environment, and interpreter. It visualizes the flow of information from each component, providing observations (states) and rewards for the agent’s decision-making process.

A reinforcement learning task comprises two key components: an “Agent” and an “Environment”, interacting as modeled by the Markov Decision Process (MDP), as shown in Figure 1. In an MDP, the role of the agent is to interact with the environment at discrete time steps t = 1, 2, 3, …, where at each time step t, the environment receives an action $A_{t}$ and returns the state of the environment $S_{t\;}\in S$ and a scalar feedback reward $R_{t}\in R$ back to the agent. The agent follows a stochastic policy π characterized by a probability distribution $\pi \left(a|s\right)=P\left[A_{t}=a|S_{t}=s\right]$ to choose an action $A_{t}\in A$. The execution of the action pushes the environment into a new state $S_{t+1}$ and produces a new scalar reward $R_{t+1}$ at the next time step t+1 according to a state transition probability $P_{ss^{\prime }}^{a}=p\left(s^{\prime },r|s,a\right)=P\left[S_{t+1}=s^{\prime },R_{t+1}=r\;|\;S_{t}=s,A_{t}=a\right]$. The primary goal of the agent is typically to find the optimal policy that maximizes the total discounted reward, which is also defined as the expected return $G_{t}=\sum_{k=t}^{\infty }\gamma^{\left(k-t\right)}R_{k+1}$, where *γ* $\in \left[0,1\right]$ is the discount factor.

However, the agent typically cannot directly interact with the environment and requires the assistance of an interpreter to mediate. The interpreter’s role is to transform raw environmental states into a format compatible with the agent and process the actions received from the agent into commands that can affect the elements in the environment. Therefore, from the agent’s perspective, the interpreter and environment together form the effective “RL environment,” replacing the abstract “environment” in the classical MDP formulation. In robotics, this process involves mapping observations and actions to their real-world or simulated counterparts, ensuring that the RL agent sends actuator commands to control the robots and receives accurate sensor readings as observations from the real world or simulation.

In most robot-based learning tasks, the state of the environment is not fully observable, and the agent relies on real-time sensor data to partially observe the environment using the observation vector $o_{t}$. The observation space generally contains continuous values, as it usually holds perception and sensory data. Therefore, most robot-based learning tasks incorporate deep learning techniques as function approximators in conjunction with reinforcement learning (also known as deep reinforcement learning, or DRL) to effectively manage continuous state spaces. In traditional RL, these techniques and methods typically center around learning the optimal policy by evaluating action values, as in the Deep Q-Network (DQN) [23], or directly parameterizing the policy with neural networks and optimizing them, as in the Twin Delayed Deep Deterministic Policy Gradient (TD3) [24] algorithms. Currently, various third-party frameworks provide libraries for state-of-the-art DRL algorithms. These frameworks provide clean, robust code with simple and user-friendly Application Programming Interfaces (APIs), enabling users to experiment with and monitor the learning of various DRL algorithms. Therefore, if users do not benchmark their custom algorithms, they can employ the functionality of these packages, such as Stable Baselines3 (SB3) [25], Tianshou [26], CleanRL [27], or RLlib [28], to effectively train robotic agents.

### 2.2. Applying Reinforcement Learning to Real-World Robots

Training real robots directly using reinforcement learning from scratch can be challenging, and most of the time, it is not actively considered due to the random nature of the exploration of the RL algorithms [29]. Initially, the agent must explore the environment thoroughly to collect data, which often requires a certain level of randomness in the agent’s actions. However, as learning progresses, the agent’s actions should become less random and more consistent with each iteration [8]. This is vital to ensure that agents do not exhibit unexpected or unpredictable behavior when deployed in the real world. Nevertheless, with proper safety measures, handpicked learning parameters, and well-defined observations, actions, and rewards, it is possible to train a robot directly in the real world [30,31]. Furthermore, incorporating techniques such as Curriculum Learning [32], where the agent maneuvers through complex tasks by breaking them down into simpler subtasks and gradually increasing the task difficulty during learning, can help train robots directly without a simulation environment [33].

While it is possible to learn directly with real robots, several constraints exist when applying RL to real-world learning tasks. One of the significant challenges is ensuring the safety of the robot, its platform, and the surrounding environment. Due to the arbitrary nature of the initial learning stage, where the agent attempts to learn more about the environment through random actions, the robot can potentially damage itself and nearby expensive equipment. Another challenge is the sample inefficiency in real-world RL environments. Most RL-based algorithms require a large number of samples to determine the optimal policy. However, unlike in a digital simulation model, it is difficult for real-world RL environments to provide fast and nearly unlimited numbers of samples for the agent to learn. This is especially apparent in episodic tasks that require environmental resetting at the end of each episode. This process is relatively straightforward in a digital model, where the environment can be reset via the API of the physics simulator and a function that programmatically sets the initial conditions of the environment. However, in the real world, this is a tedious task, where an operator may often have to constantly monitor and physically rearrange the environment at the end of each training episode. Similarly, even if it is possible to speed up learning by using an environment vectorizing approach for sampling experience with multiple concurrent environment instances, the constant monitoring and associated costs may render it infeasible for many robotics tasks.

### 2.3. Use of Simulation Models for Robotic Reinforcement Learning

Simulation-based robot learning begins by establishing a programmatic interface with a physics simulator [34] to interact with sensors and actuators to read sensory data and execute actuator commands. These types of interfaces enable the creation of RL environments that follow the conventional reinforcement learning architecture, as illustrated in Figure 1. One of the most effective structures for creating RL environments was introduced by OpenAI Gym [35]. It has become a widely adopted standard for RL-based environment creation, and a significant portion of research in the field follows a variation of this standard or builds upon the Gym package for different robotic and physics simulators. Currently, a plethora of RL simulation frameworks [36] are available for robotic task learning and typically provide prebuilt environments or tools to create custom environments. Prebuilt learning tasks are primarily used for benchmarking new learning algorithms and have been extensively employed by researchers to demonstrate RL approaches in robotics. The popularity of these learning tasks is due to their ability to relieve users from many task setup details, such as defining the observation space, action space, and reward architecture. However, custom environment creation is generally more challenging, as it requires users to become familiar with the API of the RL simulation framework and task setup details, including providing a detailed description of the robot and its surrounding environment in a format [37] that is compatible with the chosen simulator. Once the environment is created, users can utilize third-party RL library packages, such as SB3, or custom learning algorithms to find the optimal policy for the custom learning task.

## 3. Related Work

Most RL-based simulation frameworks for robots are built on simulators such as MuJoCo [38], PyBullet [39], and Gazebo [40], which prioritize accelerated simulations for developing complex robotic behaviors, often with less emphasis on the seamless transition of policies to real-world robots. A recent advancement in this field is Orbit [41] (Now Isaac Lab), a framework built upon Nvidia’s Isaac Gym [42] to provide a comprehensive modular environment for robot learning in photorealistic scenes. It is distinguished by its extensive library of benchmarking tasks and capabilities that potentially ease policy transfer to physical robots with ROS integration. However, at the current stage, its focus remains mainly on simulation rather than direct real-world learning. Although it provides tools for simulated training and real-world applications, it may not yet serve as a complete solution for real-world robotics learning without additional customization and system integration efforts. Furthermore, the high hardware requirements (https://docs.isaacsim.omniverse.nvidia.com/latest/installation/requirements.html, accessed on 17 June 2025) of Isaac Sim may restrict accessibility for many researchers and roboticists, limiting its widespread adoption.

SenseAct [9] is a notable contribution that highlights the challenges of real-time interactions with the physical world and the importance of sensor-actuator cycles in realistic settings. They proposed a computational model that utilizes multiprocessing and threading to perform asynchronous computations between the agent and the real environment, aiming to minimize the delay between observing and acting. However, this design is primarily tailored for single-task environments and shows limitations when extended to multi-robot/task research, including learning together with simulation frameworks or concurrently in multiple environments. This limitation partly stems from its architecture, which allocates a single process with separate threads for agents and environments. The scalability of this approach, particularly for concurrent learning with multiple RL environments, is hindered by Python’s Global Interpreter Lock (GIL) (https://wiki.python.org/moin/GlobalInterpreterLock, accessed on 17 June 2025), which restricts parallel execution of CPU-intensive tasks. Hence, incorporating multiple RL environment instances within a single process is not computationally efficient, especially when real-time interactions are critical. Furthermore, the difficulty in synchronizing different processes and establishing communication layers with various robots and sensors from different manufacturers may limit the potential of their proposed approach.

Table 1 provides a comprehensive comparison between UniROS and existing RL frameworks, focusing on ROS integration, real-time capabilities, and multi-robot support. Unlike most prior tools, which are either simulation-centric or designed for single-robot real-world use, UniROS is uniquely positioned to support scalable and low-latency training across both simulation and physical robots concurrently.

In addition to comprehensive frameworks, several studies have addressed specific aspects of bridging simulation and real-world robot learning. Many domain randomization approaches [43,44] either dynamically adjust the simulation parameters based on real-world data or vary the simulation parameters to improve the sim-to-real transfer. However, their methods often require extensive manual tuning of randomization ranges and do not address the fundamental timing mismatches between simulations and real-world execution. While other domain adaptation approaches [45,46], leverage demonstrations in both simulation and real-world settings to accelerate robot learning, their approach requires separate implementations for each domain. As these approaches do not provide a unified interface for concurrent learning across simulated and real environments, they highlight the need for more efficient frameworks that can leverage both simulated and real-world data concurrently.

## 4. Learning Across Simulated and Real-World Robotics Using UniROS

This section provides a high-level overview of the proposed UniROS framework formulation, which facilitates learning in both simulated and real-world domains. The aim is to present a comprehensive overview of the framework architecture and functionalities, setting the stage for a more detailed examination of its components in subsequent sections.

### 4.1. Unified Framework Formulation

**Figure 2:**
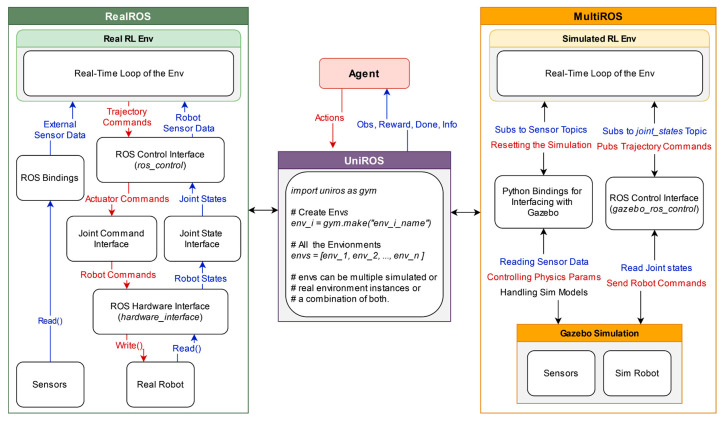
This diagram illustrates the interconnected components of the proposed system, highlighting the dual-package approach with RealROS for real-world robotic applications and MultiROS for simulated environments. The UniROS layer acts as an abstraction to standardize the communication process, facilitating the creation and management of multiple environmental instances. The diagram encapsulates the ROS-based data flow and primary interfaces for robot control, which handles real-time interactions.

As illustrated in Figure 2, a ROS-based unified framework is proposed, which contains two distinct yet interoperable packages to bridge the learning across simulated and real-world RL environments. We previously introduced MultiROS [47], which provides simulation environments using ROS and Gazebo as its core. In contrast, the newly introduced RealROS package, which is detailed in Section 5, was designed explicitly for real-world learning applications. The intuition behind dividing the framework into two packages is to offer users flexibility. Depending on specific requirements, users can utilize each package independently, focusing solely on either simulated or real-world scenarios, or leverage them collectively for comprehensive simulation-to-reality learning tasks. Furthermore, Section 7 presents an ROS-centric RL environment implementation strategy to bridge the learning gap between the two domains. It aligns the conditions and dynamics of the Gazebo simulation more closely with those of the real world, allowing for a smoother deployment of policies from simulation to the real world. This environment implementation strategy can also be employed with the RealROS package to develop and deploy robust policies by sampling directly from real-world environments without relying on a simulated approach.

### 4.2. Modularity of the Framework

The architecture of both MultiROS and RealROS leverages a modular design by segmenting the creation of the RL environment into three distinct classes. The primary focus of this segmentation is to enable flexibility and encourage efficient code reuse during the design of RL environments. These segmented environment classes (*Env*) provide a structure for users to format their code easily and minimize system integration efforts when transferring policies from the simulation interface to the real world. Therefore, this framework provides an architecture comprising three major components for delivering these services. These are (A) the *Base Env*, the foundational layer of the RL environment, which facilitates the main interface of the standard RL structure for the agent. It inherits its core functionality from OpenAI Gym and includes static code essential for the basic functioning of any RL task. (B) The *Robot Env*, which is built on the *Base Env*, outlines the sensors and robots used in the learning task. It encapsulates all the hardware components involved in the task and serves as a bridge that connects the RL environment with the ROS middleware suite. (C) The *Task Env* extends from the *Robot Env* and specifies the structure of the task that the agent must learn, which includes learning objectives and task-specific parameters. These modular *Envs* provide a significant degree of flexibility, allowing users to create multiple *Task Envs* with a single *Robot Env*, as with the Fetch environments (https://robotics.farama.org/envs/fetch/index.html, accessed on 17 June 2025) (*FetchReach*, *FetchPush*, *FetchSlide*, and *FetchPickAndPlace*) of OpenAI Gym robotics. It is important to note that while each *Task Env* is compatible with its respective *Robot Env* (which may include multiple robots and different sensors), they are not universally interchangeable across different *Robot Envs*. Therefore, modularity is used for diverse task development based on a specific *Robot Env*. The main reason for this composition is that *Base Env* inherits from OpenAI Gym to retain the compatibility of third-party reinforcement learning frameworks such as Stable Baselines3, RLlib, and CleanRL.

### 4.3. Python Bindings for ROS

One of the drawbacks of ROS is that some crucial system-level components required to create RL environments do not have Python or C++ bindings. For example, executing and terminating ROS nodes or launch files requires terminal commands (CLIs). Similarly, running multiple roscore instances concurrently using Python or C++ interfaces and managing communication with each other is currently not natively supported in ROS. These functions also require command-line interfaces, making them undesirable for seamless RL environment creation. Therefore, the UniROS framework contains comprehensive Python-based bindings for ROS, enabling users to utilize the full potential of ROS for creating RL environments. Key features include the ability to launch multiple ROScores on distinct or random ports without overlap, manage simultaneous communication between concurrent ROScores, run roslaunch and ROS nodes with specific arguments, terminate specific ROS masters, nodes, or roslaunch processes within an environment, retrieve and load YAML files from a package to the ROS Parameter Server, upload a URDF to the parameter server, and process URDF data as a string.

### 4.4. Additional Supporting Utilities

In addition to the stated ROS bindings, the framework also provides utilities based on Python for users to quickly start creating environments without wasting too much time on ROS implementations. It is also beneficial for users unfamiliar with ROS to create environments without expert knowledge of programming with ROS. These utilities include the ROS Controllers module, which allows comprehensive control over ROS controllers to load, unload, start, stop, reset, switch, spawn, and unspawn controllers. The ROS Markers module provides methods for initializing and publishing Markers or Marker arrays to visualize vital components of the task, such as the current goal, pose of the robot’s end-effector (EE), and robot trajectory. This makes it easy to monitor the status of the environment using Rviz (http://wiki.ros.org/rviz, accessed on 17 June 2025), a 3D visualization tool for ROS, to visualize the markers. The ROS kinematics module provides forward (FK) and inverse kinematics (IK) functionalities for robot manipulators. This class uses the KDL (https://www.orocos.org/kdl.html, accessed on 17 June 2025) library to perform kinematics calculations. The MoveIt Module offers essential functionalities for managing ROS MoveIt [48] in manipulation tasks, including collision checking, planning, and executing trajectories. Additionally, common wrappers are included to limit the number of steps in the environment and normalize the action and observation spaces.

The utilities and other functionalities introduced here are not limited to creating RL environments and are equally valuable for any ROS-based workflow that must couple simulation and hardware through sensorimotor inferences. One of the prime examples is ROS-centric digital twin pipelines, where ROS’s publish/subscribe architecture mediates communication between the virtual twin and its real-world counterpart. Singh et al. [49] have already shown that the proposed functionality of UniROS further simplifies this process for interacting with multiple real-world and simulation instances with minimal effort.

The complete source codes of MultiROS (https://github.com/ncbdrck/multiros, accessed on 17 June 2025) and RealROS (https://github.com/ncbdrck/realros, accessed on 17 June 2025), along with the necessary supporting materials (https://github.com/ncbdrck/uniros_support_materials, accessed on 17 June 2025), are currently available online to the robotics community as public repositories. They accompany well-documented templates, guides, and examples to provide clear instructions for their installation, configuration, and usage.

## 5. An In-Depth Look into ROS-Based Reinforcement Learning Package for Real Robots (RealROS)

**Figure 3:**
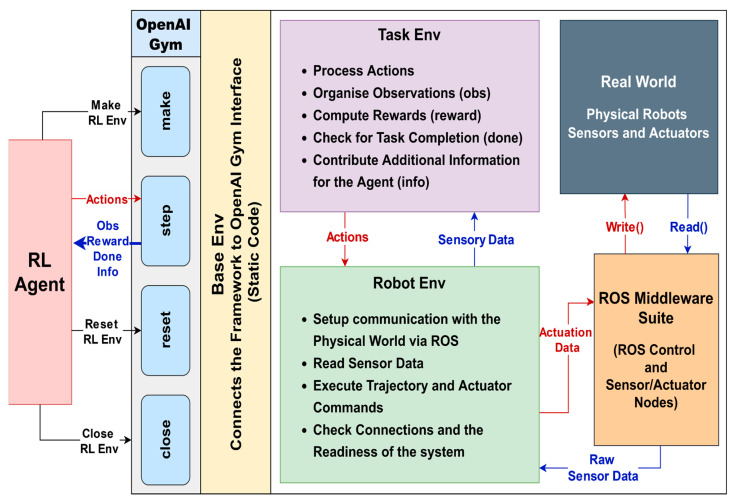
The architecture of the proposed RealROS provides a modular structure inherited from the OpenAI Gym. The *Task Env* inherits from the *Robot Env*, which, in turn, inherits from *Base Env*, and *Base Env* inherits from the OpenAI Gym. An environment instance created with this architecture only exposes the standard RL structure to the agent, allowing it to accept actions, pass them through the *Robot Env* from the *Task Env*, and execute them in the real world. Then, the *Task Env* obtains observations from the methods defined in the *Robot Env*, calculates the Reward and Done flags, and returns them to the agent.

This section describes the overall system architecture of the RealROS package. It is designed to be compatible with the MultiROS simulation package and is structured to minimize the typical extensive learning curve associated with switching from simulation to a real environment. The three core components (*Base Env*, *Robot Env*, and *Task Env*) provide the main API for the experimenters to encapsulate the task, as illustrated in Figure 3. The integration of the stated attributes and the structure of the three main components of the framework are described in detail in the following sections.

### 5.1. Base Env

*Base Env* serves as the superclass that provides the foundation and main interface that specifies the standard RL structure for RealROS. It provides the necessary infrastructure and other essential components for RL agents to interact with their environment. RealROS offers two options for users to create environments. The default “standard” (*RealBaseEnv*) is based on the inheritance of gym.Env, and the other “goal-conditioned” (*RealGoalEnv*) is from the gym.GoalEnv of the OpenAI Gym package. This *Base Env* defines the step, reset, and close methods, which are the main standardized interface of OpenAI Gym-based environments. When initializing the *Base Env*, experimenters can pass arguments from the *Robot Env* to perform several optional functions based on the user’s preferences. These include initiating communication with real robots, changing the current ROS environment variables, loading ROS controllers for robot control, and setting a seed for the random number generator.

### 5.2. Robot Env

*Robot Env* is a crucial component for describing robots, sensors, and actuators used in real-world environments using the ROS middleware suite. It inherits from the *Base Env*, allowing it to initialize and access methods defined in the superclass. However, it adds additional functionalities that are specific to robots and sensors used in the real world. This *Robot Env* encapsulates the following aspects.

Robot Description: One of the primary tasks of the *Robot Env* is to define the physical robot characteristics, such as its kinematics, dynamics, and available sensors, using a format that ROS can understand. For this purpose, the ROS requires the robot description to be loaded into the ROS Parameter Server (http://wiki.ros.org/Parameter%20Server, accessed on 17 June 2025) (a shared, multi-variate dictionary that is accessible via ROS network APIs). In ROS, the robot description is in the Universal Robot Description Format (URDF (http://wiki.ros.org/urdf, accessed on 17 June 2025)) and contains all relevant information about the composition of the robot. These include joint types, joint limits, link lengths, and other intrinsic parameters of the robot. By loading the robot description, ROS packages can utilize it to perform collision detection, inverse kinematics (IK), and forward kinematics (FK) calculations of the robot arm. Suppose that multiple robots are required for the learning task (inside the same RL environment). In this case, each robot’s description can be loaded with a unique ROS namespace identifier to distinguish it from the others.

Set up communications with robots: Currently, most robot manufacturers or the ROS community typically provide essential ROS packages specifically designed for commercial robots to establish communication channels with their robots. One of the prime examples is the ROS-Industrial (https://rosindustrial.org/, accessed on 17 June 2025) project, which extends the advanced capabilities of ROS for industrial robots from manufacturers (http://wiki.ros.org/Industrial/supported_hardware, accessed on 17 June 2025), such as ABB, Fanuc, and Universal Robots. These packages include the robot controller software, which is responsible for managing the robot’s motion and maintaining communication with the robot’s motors and sensors, as well as with external systems. For custom robots, the official ROS tutorials (http://wiki.ros.org/ROS/Tutorials, accessed on 17 June 2025) provide details on creating a custom URDF file and ROS controllers (http://wiki.ros.org/ros_control/Tutorials, accessed on 17 June 2025) for interfacing with physical hardware. Once these packages are properly configured, the RL environment can send commands to control the robot’s motors and access its sensor readings (including motor encoders) through ROS.

Setting up communication with the Sensors: Similarly, connecting external sensors using ROS enables the acquisition of data from various sensors, such as cameras, lidars, proximity sensors, and force/torque sensors. This can help portray a vivid picture of the real world, enabling the RL agent to perceive the current state of the environment (i.e., observations). Currently, most vision-based sensors (https://rosindustrial.org/3d-camera-survey, accessed on 17 June 2025) and others (http://wiki.ros.org/Sensors, accessed on 17 June 2025) have ROS packages provided by the manufacturers or the ROS community, allowing users to easily plug and play the devices. Typically, raw data from these components must be converted into a format that the RL agent can process. As most agents are implemented using deep learning libraries such as PyTorch, TensorFlow, or JAX, these image and sensor data are typically transformed into NumPy arrays or other compatible formats.

Robot Env-specific methods: These methods are for the RL agent to interface with robots and other equipment in the environment. They can be used to plan trajectories, calculate IK, and control the joint positions, joint forces, and speed/acceleration of the robot. These methods are then used in the *Task Env* to execute the agents’ actions in the real world. Furthermore, ROS’s inbuilt utilities and other packages of ROS, such as MoveIt, provide functionality for obtaining the transformations of each joint (tf (http://wiki.ros.org/tf, accessed on 17 June 2025)) and the current pose (3D position and orientation) of the robot end-effector to portray the current state of the robot. Combining these with the data acquired from custom methods for interfacing with external sensors enables the *Task Env* to construct observations of the environment.

### 5.3. Task Env

The *Task Env* serves as a module that outlines the structure and requirements of the task that the RL agent must learn. It inherits from the *Robot Env* and builds upon its functionalities to create a real-time loop (Section 7) that executes actions, constructs observations, calculates the reward, and checks for task termination (optional). Therefore, *Task Env* defines the observation space, action space, goals or objectives, rewards architecture, termination conditions, and other task-specific parameters. These components help with the main *Step* function of the environment to take a step in the real world and send feedback (observations, reward, done, and info) to the agent. Furthermore, knowledge acquired in the simulation (MultiROS) can be efficiently transferred to real-world environments by reusing the same code to create the Task Env, owing to the modular design of the UniROS framework and the compatibility between the MultiROS and RealROS packages, with the caveat that the new *Robot Env* must implement the same helper functions expected by the *TaskEnv*.

Table 2 summarizes the core components, including their function-level responsibilities for each Env. This modular interface design enables both reuse and extension across robots and tasks while maintaining consistency in the learning loop.

## 6. ROS-Based Concurrent Environment Management

This section discusses how the UniROS framework sets up ROS-based concurrent environments to maintain seamless communication. First, it deliberates the challenges and provides solutions for initiating multiple ROS-based simulated and real-world environment instances inside the same script. Subsequently, it describes the steps for launching multiple Gazebo simulation instances and connecting real-world robots over local and remote connections while ensuring that each environment operates independently and interacts effectively without interference.

### 6.1. Launching ROS-Based Concurrent Environments

Launching OpenAI Gym-based concurrent environments presents several unique challenges when working with ROS. One challenge is the functionality of the OpenAI Gym interface, which executes all the environment instances within the same process when launched using the standard gym.make function. In contrast, ROS requires each environment instance (sim or real) or process to initialize a unique ROS node (http://wiki.ros.org/Nodes, accessed on 17 June 2025) to utilize ROS’s built-in functions and communicate with the corresponding sensors and robots via the ROS middleware suite. However, ROS typically does not support the initialization of multiple nodes inside the same Python process due to its fundamental design architecture, which is centered around process isolation for enhanced reliability, modularity, and robustness. Therefore, launching multiple ROS-based environments within the same script is challenging because it typically requires initializing separate ROS nodes for each instance. Although there is a *roscpp* (http://wiki.ros.org/roscpp, accessed on 17 June 2025) (C++ client of ROS) feature for launching multiple nodes inside the same script called *nodelet* (http://wiki.ros.org/nodelet, accessed on 17 June 2025), the Python client *rospy* (http://wiki.ros.org/rospy, accessed on 17 June 2025) does not currently support this function.

One workaround is to employ Python multi-threading to launch each RL environment instance, allowing the execution of multiple ROS nodes within the same process. However, this can lead to unexpected behaviors, particularly in terms of efficiently utilizing computational resources and parallel processing between instances. One limiting factor of multi-threading is that Python’s Global Interpreter Lock (GIL) prevents multiple threads from executing Python bytecodes simultaneously. This is unfavorable for working with concurrent environments, as each environment instance requires CPU-intensive real-time agent-environment interactions, as highlighted in Section 7.

Therefore, the UniROS framework utilizes a Python multiprocessing wrapper on the OpenAI Gym interface to launch each environment instance as a separate process to address these limitations. Initializing separate processes for each environment instance also launches a separate Python interpreter for each process, allowing them to overcome the bottleneck imposed by the Python GIL. Process isolation can significantly contribute to optimizing resource allocation for efficiently performing parallel computations with each environment instance. For users, this transition is virtually effortless, as they need to utilize UniROS, as in Figure 2, instead of the standard gym.make function to launch environments. This adaptation can effortlessly leverage optimized parallel processing capabilities that are crucial for real-time CPU-intensive computation tasks in ROS-based concurrent environments.

### 6.2. Maintaining Communication with Concurrent Environments

**Figure 4:**
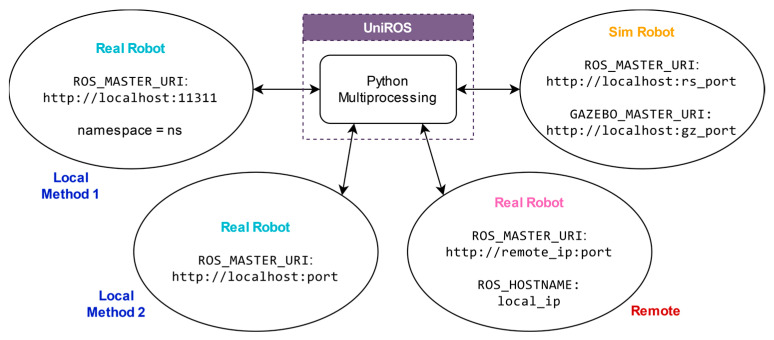
Communication Schemes of the UniROS Framework for Concurrent ROS-Based Environments. The diagram presents (1) two methods for local connections, utilizing either namespaces or separate ROScores for enhanced process isolation; (2) a configuration employing ROS multi-device mode for robust remote communication; and (3) a configuration for Gazebo-based simulations that enable concurrent environments.

A robust communication infrastructure is essential for managing concurrent environments to ensure seamless interaction between multiple real robots and Gazebo simulation instances. Figure 4 outlines the methodologies employed in the UniROS framework to facilitate such communication across simulated and real environments. In MultiROS-based simulation environments, this was achieved by launching an individual roscore (http://wiki.ros.org/roscore, accessed on 17 June 2025) for each environmental instance. In ROS, roscore provides necessary services such as naming and registration to a collection (graph) of ROS nodes to communicate with each other. Without a roscore, nodes are unable to locate each other, exchange messages, or establish parameter settings, making it a critical component in ensuring coordinated operations and data exchange within the ROS ecosystem. Therefore, by utilizing separate ROScores, each roscore acts as a server and creates a dedicated communication domain for each node (client) within the environment instance to exchange data without cross-interference from other concurrent instances. Therefore, for simulations, MultiROS utilizes separate ROScores and dynamically assigns the environment variables mentioned in Figure 4 inside each simulated environment, allowing for precise management of interactions and data flow across concurrent environments. Here, the ROS Python bindings of the UniROS framework were employed to launch ROScores with non-overlapping ports and Gazebo simulations, as well as functions for handling ROS-related environment variables to streamline the process without relying on CLI configurations.

For real-world learning tasks, the RealROS package facilitates robot connections using both local and remote methods. In local setups, the default configuration typically involves using the standard roscore, which uses port ‘*11311*’ as the ROS master (http://wiki.ros.org/rosmaster, accessed on 17 June 2025) by default. In such arrangements, each robot and its corresponding sensors can be assigned a distinct namespace to distinguish them from other robots within the same shared roscore. This unique namespace can be used in the respective environment to ensure orderly and isolated interactions, despite the shared communication space with other robots. However, for scenarios requiring enhanced communication isolation, each robot can be configured to connect to different ROScores, each operating on a unique, non-overlapping ROS master port. Then, setting the appropriate *ROS_MASTER_URI* environment variable in each environment instance adds an extra layer of process isolation to effectively eliminate the potential for cross-interference, which is typically inherited with namespace-based setups.

In remote configurations where robots are connected to the same network, robots can be connected in ROS multi-device mode, where the local PC functions as a client capable of reading and writing data to the remote server (robot) that runs the master roscore. This setup eliminates the need for Secure Shell (SSH) approaches and allows all the processing required for learning to be conducted on the local PC. This approach is particularly beneficial for robots created for research, where the robot’s ROS controller is based on less powerful edge devices, such as the Raspberry Pi, Intel NUC, and Nvidia Jetson. This ROS multi-device mode can be configured by setting the environment variables on both the robot and the local PC, as depicted in Figure 4. This setup allows for multiple concurrent environments, each connecting to a remote robot by setting the ROS environment variables using the Python ROS binding of the UniROS framework. This approach ensures a clear distinction between each remote robot, facilitating organized communication across concurrent environments.

Using these methodologies, the UniROS framework can efficiently manage multiple concurrent environments, whether they are purely simulated robots, real robots, or a combination of both. Furthermore, this study provides ready-to-use templates within the respective MultiROS or RealROS packages to create and manage RL environments, eliminating the need to handle these ROS Python bindings directly. These templates, including the respective *Base Env* of the packages, relieve experimenters from explicitly handling ROS system-level configurations, allowing them to focus more on implementing their learning tasks.

## 7. Setting Up Real-Time RL Environments with the Proposed Framework

This section outlines the essential components required for establishing real-time learning tasks using ROS native components. It explores various options for each component and details specific recommendations for implementing real-time RL environments using the UniROS framework. The ROS-based environment implementation strategy described here ensures that simulated environments closely mirror real-world scenarios for seamless policy transfer and that real-world environments are safe and effective in facing the challenges of real-world conditions and dynamics.

### 7.1. Overview of the Real-Time Environment Implementation Strategy

One key challenge in real-time learning tasks is that sensory data and control commands are typically processed sequentially, which inherently introduces latency. This latency results from the time it takes the environment to construct observations and rewards and for the agent to decide on the subsequent action. Because all calculations in the typical MDP architecture are performed sequentially, this can lead to a misalignment between the state of the environment and the agent’s perception of it. Therefore, it makes the learning problem more complex and may push the agent to develop suboptimal strategies simply because it reacts to outdated information. From the agent’s perspective, augmenting the state space with a history of actions may help the agent anticipate and adapt to delays. However, these methods do not reduce the actual latency of the system but merely help the agent cope with it [50].

**Figure 5:**
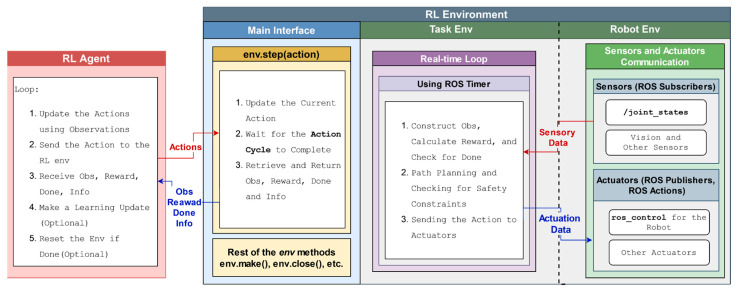
Diagram of a real-time environment implementation strategy for RL with ROS integration, detailing the interaction between the RL Agent process and the RL Environment process. The agent process updates actions based on observations and refines its policy, while the environment loop thread of the environment process handles observation creation, reward calculation, and sending actuator commands, all in real time. This design minimizes latency, allowing for dynamic interactions between the RL agent and environment in both simulation and real-world learning tasks.

Therefore, this study proposes an asynchronous scheduling approach for agent-environment interactions, allowing concurrent processing to minimize overall system latency. This design partitions the reinforcement learning agent and environment into two distinct processes, as depicted in Figure 5. In the environment process, multi-threading is employed to concurrently read sensor data and send the robot actuator commands via the sensorimotor interface to avoid unnecessary system delays. Similarly, an additional dedicated environment loop thread is used to periodically oversee the construction of observations, the calculation of rewards, and the verification of task completion. It also iteratively performs safety checks and updates actuation commands in response to the agent’s actions in real time. The intuition behind employing an environmental loop is to allow the agent to make decisions and send actions without waiting for sensor readings or actuator commands to be processed, which helps minimize agent-environment latency.

Conversely, the RL agent process updates the actions using observations and performs learning updates to refine its policy. It also defines the task-specific duration between successive actions (action cycle/step size), which is a hyperparameter that sets the rate of agent-environment interactions. This computational model of the framework is scalable for learning scenarios that require multiple concurrent environments by utilizing one process per environment, as described in Section 6.1, enabling the agent to use multi-robot/task learning approaches. Here, the proposed approach does not use multi-threading packages of Python and, instead, utilizes the offerings of ROS to implement the proposed real-time RL environment implementation strategy. The primary reason for this selection is the specialized capabilities of ROS, which offer optimized low-latency communication and a flexible, scalable architecture that is better suited for complex robotic systems. These ROS primitive features have been widely validated in various real-world applications and are well-suited for scaling to real-time multi-robot reinforcement learning setups. Furthermore, this abstraction not only reduces integration complexity for developers but also ensures broad compatibility with a wide variety of commercial and research robots that already support ROS.

Therefore, with this approach, the agent and the ROS-integrated environments run as separate Python processes. Here, the agent communicates with the environment via the standard Gym-based interface (*env.step()*, *env.reset()*), while the RL environment manages ROS-based communication internally. This allows the RL agent to remain agnostic to ROS-specific details belonging to the RL environment setup, enabling seamless integration with standard RL libraries, such as Stable-Baselines3 and others.

### 7.2. Reading Sensor Data with ROS

In the ROS framework, sensor packages typically employ ROS Publishers to publish data across various ROS topics (http://wiki.ros.org/Topics, accessed on 17 June 2025). For instance, the *joint_state_controller* (http://wiki.ros.org/joint_state_controller, accessed on 17 June 2025) of the *ros_control* (http://wiki.ros.org/ros_control, accessed on 17 June 2025) package launches a ROS node, which is essentially a separate process dedicated to continuously monitoring the robot’s joint positions, velocities, and efforts based on sensor (joint encoders) feedback. This node then periodically publishes captured data to the *joint_states* topic, allowing other packages to monitor the robot’s status by subscribing to it. The ROS Subscriber is a built-in ROS component that listens to messages on a specific topic, which triggers a callback function to handle the incoming data each time a message is published on the topic. Because the handling of subscriber callbacks in *rospy* (Python client library for ROS) is inherently multi-threaded, each sensor callback is handled in a separate thread, allowing for the concurrent processing of sensor data from different topics. This approach is adaptable and can be extended to integrate additional sensors, such as a Kinect camera (RGBD), into ROS using respective sensor packages. These packages are responsible for interfacing with the sensor hardware, processing the data, and publishing it to relevant ROS topics, which can be used as part of the observations to provide a comprehensive overview of the environment. However, it should be noted that while the Python GIL does not hamper I/O-bound operations such as ROS subscribers, it can cause a bottleneck if the callback function contains CPU-intensive operations (e.g., preprocessing camera data for object detection). In such scenarios, using a separate ROS node that creates a separate process may be beneficial for handling CPU-intensive computations.

### 7.3. Sending Actuator Commands with ROS

In real-time learning tasks, controlling the robot by directly accessing the low-level control interface of ROS is required instead of relying on high-level motion planning packages such as MoveIt. This choice was motivated by the need for real-time control of the trajectories, which is a key feature that is better managed by the robot’s low-level controllers. This approach provides the flexibility necessary for dynamic behaviors, such as smooth and rapid switching between trajectories. While MoveIt is excellent for planning and executing trajectories, its higher level of abstraction limits its capability for instant preemption of trajectories and swift adjustments while maintaining a continuous motion. Therefore, this implementation strategy bypasses MoveIt and directly interacts with the controllers by publishing to the respective *ros_control* topic since real-time learning requires rapid trajectory updates.

### 7.4. Environment Loop

The environment loop is the cornerstone of the proposed RL environment implementation strategy, which facilitates the interaction between the RL agent and the robotic environment. It manages the timing and synchronization to ensure seamless integration between the agent’s decision-making process and the robot’s trajectory execution. This loop is implemented using ROS timers, a built-in feature of ROS that enables the scheduling of periodic tasks. One significant advantage of this method is that the callback function for the ROS timer is executed in a separate thread, similar to the callbacks of the ROS Subscribers. Therefore, this approach ensures that the computational load of other threads (mostly I/O-bound) does not interfere with the performance of the environment loop.

Hence, the proposed implementation strategy initializes a ROS timer for the environment loop to trigger at a fixed frequency (environment loop rate) throughout the entire operation runtime to execute the sequence of operations, as shown in Figure 5. At each trigger, it captures the current state of the environment through data acquired from sensors and formalizes them into observations. Then, it calculates the reward and assesses whether the conditions for a done flag are met to indicate the end of the episode. Finally, it checks for safety constraints and executes the last action received from the RL agent. When executing actions, it repeats the previous action if no new action is received during the next timer trigger. This approach, also known as action repeats [23], reduces the agent’s computational load and allows for smoother and more stable manipulations as the robot maintains its course of action for a consistent duration. Typically, the environment loop rate is several times higher than the action cycle time (step size) of the robot. Therefore, every time the agent process sends an action, the environment process does not need to wait to process the relevant returns (observations, reward, done, and info) after the step size passes because it can retrieve the latest returns already constructed in the environment loop and pass them to the agent.

The environment loop rate is a hyperparameter used to configure the real-time environment implementation. However, it is essential to mention that each robot has its own hardware control loop frequency, which determines how quickly it can process information, execute relevant control commands, and read sensor (joint encoder) data. Therefore, setting the environment loop rate higher than the hardware control loop frequency of the robot is counterintuitive, as the robot cannot physically execute control commands or retrieve its status at a higher frequency than its hardware control loop allows. This hardware control loop frequency depends on the robot’s capabilities and is typically set by the manufacturer in the *hardware_interface* (http://wiki.ros.org/hardware_interface, accessed on 17 June 2025) of the robot’s ROS controller.

The ROS Hardware Interface of a robot is responsible for reading the sensor data, updating the robot state via the *joint_states* topic, and issuing commands to the actuators. This loop runs continuously during the entire robot operation and is tightly synchronized with the robot control system. Hence, operating at the hardware control loop frequency ensures that the sensor data readings and actuator command executions are closely aligned with the operational capabilities of the robot. This loop rate is usually included in the robot’s documentation or inside a configuration file (YAML file) of the robot’s ROS controller repository. However, it is also possible to select an environment loop rate lower than the robot’s hardware control loop frequency because this does not hinder the robot’s normal operation. The effects of choosing a lower or higher frequency for the environment loop rate, along with the impact of action cycle time on learning, are further discussed in Section 9 using the benchmark learning tasks introduced in Section 8.

## 8. Benchmark Tasks Creation

This section discusses the development of benchmark tasks using both MultiROS and RealROS packages. These simulated and real environments are then used in the subsequent sections to explain and evaluate the proposed real-time environment implementation strategy and to demonstrate some use cases of the UniROS framework. These tasks are modeled closely after the Reach task of the OpenAI Gym Fetch robotics environments [51], where an agent learns to reach random target positions in 3D space. In each Reach task, the robot’s initial pose is the default “*home*” position of the robot (typically set to zero for all the joint angles of the robot), and the agent’s goal is to move the end-effector to a target position $\left(x_{g},y_{g},z_{g}\right),$ to complete the task. Therefore, each task generates a random 3D point as the target at each environment reset, and the task is completed when the end-effector reaches the goal within $\varepsilon_{r}$ Euclidean distance where $\varepsilon_{r}$ (reach tolerance) is set to 0.02 m. However, unlike the Fetch environments, where the action space represents the Cartesian displacement of the end-effector, these tasks use the joint positions of the robot arm as actions. This selection was motivated by the fact that joint position control typically aligns better with realistic robot manipulations, offering enhanced precision and simpler action spaces. The following describes the details of the ReactorX 200 and NED 2 robots and the Reacher tasks (Rx200 Reacher and Ned2 Reacher) creation.

The ReactorX 200 robot arm (Rx200) by Trossen Robotics is a five-degree-of-freedom (5-DOF) arm with a 550 mm reach. It operates moderately at a hardware control loop frequency of 10 Hz. This compact robotic manipulator is most suitable for research work and natively supports ROS Noetic (http://wiki.ros.org/noetic, accessed on 17 June 2025) without requiring additional configuration or setup. It connects directly to a PC via a USB cable for communication and control, providing a reliable and straightforward method of connectivity (using the default ROS master port). All the necessary packages for controlling the Rx200 using ROS are currently available from the manufacturer as public repositories on GitHub (https://github.com/Interbotix/interbotix_ros_manipulators, accessed on 17 June 2025). Similarly, the NED2 robot by Niryo is also designed for research work and features six degrees of freedom (6 DOF) with a 490 mm reach. It has a slightly higher control loop frequency of 25 Hz and natively runs ROS Melodic (https://wiki.ros.org/melodic, accessed on 17 June 2025) on an enclosed Raspberry Pi. Niryo offers three communication options for connecting the NED2, including a Wi-Fi hotspot, direct Ethernet, or connecting both devices to the same local network. As SSH-based access was not desirable, this study opted for a direct Ethernet connection and utilized the ROS multi-device mode, as described in Section 6.2, to ensure a robust communication setup. Furthermore, Niryo also provides the necessary ROS packages (https://github.com/NiryoRobotics/ned_ros, accessed on 17 June 2025) to be installed on the local system, enabling custom messaging and service interfaces to access and control the remote robot through ROS.

Since two variants of the *Base Env* (standard and goal-conditioned) are available in this framework, two types of RL environments were created for both simulation and the real world. These simulated and real environments involve continuous actions and observations and support sparse and dense reward architectures. In the created goal-conditioned environments, the agent receives observations as a Python dictionary containing typical, achieved, and desired goals. The achieved goal is the current 3D position of the end-effector $\left(x_{a},y_{a},z_{a}\right)$, which is obtained using FK calculations, and the desired goal is the randomly generated 3D target $\left(x_{g},y_{g},z_{g}\right)$. One of the decisions made during task creation is to include the previous action as part of the observation vector, as this can minimize the adverse effects of delays on the learning process [52]. Additionally, the observation vector includes the position of the end-effector with respect to the base of the robot, the current joint angles of the robot, the Cartesian displacement, and the Euclidean distance between the EE position and the goal. Additional experimental information, including details on actions, observations, and reward architecture, is provided in Appendix A.

Furthermore, specific constraints were implemented on the operational range of both types of environments to ensure the safe operation of the robot and prevent any harm to itself or its surroundings. One of the steps taken here is to limit the goal space of the robot so that it cannot sample negative values in the *z*-direction in the 3D space. This is vital since the robot is mounted on a flat surface, making it impossible to reach locations below it. Additionally, before the agent executes the actions with the robot, the environment checks for potential self-collision and verifies whether the action would cause the robot to move toward a position in the negative *z*-direction. Therefore, the forward kinematics are calculated using the received actions before executing them to avoid unfavorable trajectories, allowing the robot to operate within a safe 3D space. Hence, considering the complexity of the tasks and compensating for the gripper link lengths, the goal space was meticulously refined to have a maximum 3D coordinates of x:0.40, y:0.40, z:0.40, and a minimum of x: 0.15, y: −0.40, z: 0.15 (in meters) for both robots.

As for the learning agents of the experiments in this study, the vanilla TD3 was used for standard-type environments and TD3 + HER for goal-conditioned environments. TD3 is an off-policy RL algorithm that can only be used in environments with continuous action spaces. It was introduced to curb the overestimation bias and other shortcomings of the Deep Deterministic Policy Gradient (DDPG) algorithm [53]. Here, TD3 was extended by combining it with Hindsight Experience Replay (HER) [54], which encourages better exploration of goal-conditioned environments with sparse rewards. By incorporating HER, TD3 + HER improves the sample efficiency because HER utilizes unsuccessful trajectories and adapts them into learning experiences. This study implemented these algorithms using custom TD3 + HER implementations and the Stable Baselines3 (SB3) library, adding ROS support to facilitate their integration into the UniROS framework. The source code and supporting utilities are available on GitHub (https://github.com/ncbdrck/sb3_ros_support, accessed on 17 June 2025), allowing other researchers and developers to leverage and build on this work. Detailed information on the RL hyperparameters used in the experiments is summarized in Appendix B. Furthermore, all computations during the experiments were conducted on a PC with an Nvidia 3080 GPU (10 GB VRAM) and an Intel i7-12700 processor with 64 GB DDR4 RAM.

## 9. Evaluation and Discussion of the Real-Time Environment Implementation Strategy

This section examines the intricacies of the proposed ROS-based real-time RL environment implementation strategy, utilizing benchmark environments as an experimental setup. The primary goal here is to discuss the two main hyperparameters of the proposed implementation strategy and gain an understanding of how to select suitable values for them, as they largely depend on the hardware capabilities of the robot(s) used in the learning task. Initially, experiments were conducted to investigate different action cycle times and environment loop rates to uncover the intricate balance between control precision and learning efficiency. Subsequently, the exploration was extended to include an empirical evaluation of asynchronous scheduling within the proposed environment implementation strategy. This process involves a thorough analysis of the time taken for each action and actuator command cycle across numerous episodes.

### 9.1. Impact of Action Cycle Time on Learning

In the real-time RL environment implementation strategy, the action cycle time (step size) is a crucial hyperparameter that determines the duration between two subsequent actions from the agent. The selection of this duration impacts the learning performance due to the use of action repeats in the environment loop. Action repeats ensure that the robot can perform smooth and continuous motion over a given period, especially when the action cycle time is longer. This technique helps to stabilize the robot’s movements and maintain consistent interaction with the environment between successive actions.

Selecting a shorter action cycle time, close to the environmental loop rate, would reduce the reliance on action repeats and enable faster data sampling from the environment due to more frequent agent-environment interactions. This would allow the agent to have finer control (high precision) over the environment at the cost of observing minimal changes. Such minimal changes can adversely affect training, as the agent may not perceive significant variations in the observations necessary for effectively updating deep neural network-based policies such as TD3. Conversely, selecting a longer action cycle time could lead to more action repeats and substantial changes in observations between successive actions, potentially easing and enhancing the learning process for the agent. However, this comes at the risk of reduced control precision and potentially slower reaction times to environmental changes, which can be detrimental in highly dynamic environments. Furthermore, this could potentially slow down the agent’s data collection rate, leading to a longer training time.

**Figure 6:**
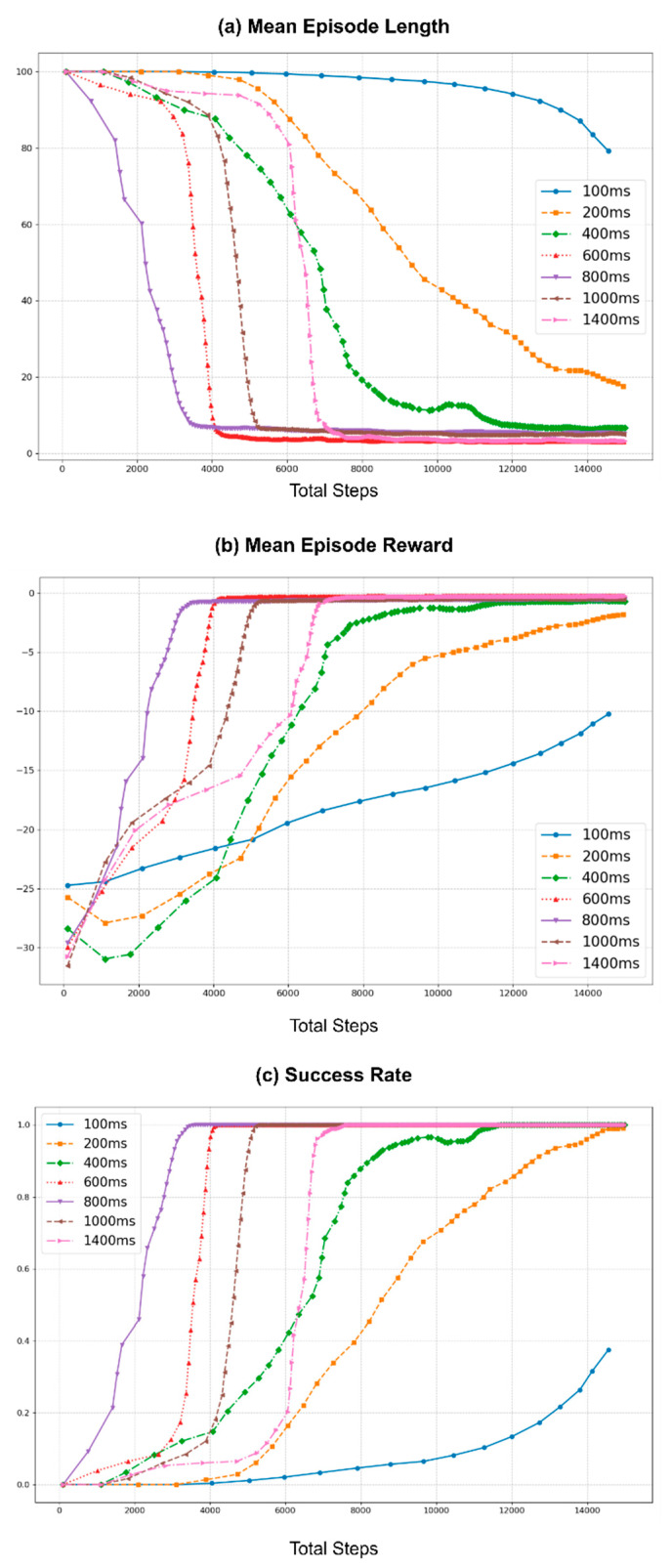
Impact of action cycle times on the learning performance of the Rx200 Reacher benchmark task. (**a**) Mean episode length, where a shorter length indicates better performance. (**b**) Mean total reward per episode, with higher rewards reflecting improved learning. (**c**) Success rate, which indicates the proportion of episodes that were successfully completed. Optimal performance is observed at action cycle times of 600 ms to 800 ms, with performance declining at longer cycle times due to reduced control precision and slower reaction times.

Therefore, to study the effect of action cycle time on learning, experiments were conducted with multiple durations, selecting a baseline, and comparing the effects of longer action cycle times, as depicted in Figure 6. These experiments were conducted in the real-world Rx200 Reacher task using the same initial values and conditions and employing the vanilla TD3 algorithm. This figure contains three graphs that illustrate the learning curves of the training process for all selected action cycle times. Figure 6a shows the mean episode length, which represents the mean number of interactions that the agent has with the environment while attempting to achieve the goal within an episode. Ideally, the episode length should be shortened over time as the agent learns the optimal way to behave in its environment. Similarly, Figure 6b depicts the mean total reward obtained per episode during training. The agent’s goal is to maximize this reward by improving its policy and learning to complete the task efficiently. Furthermore, in the benchmark tasks, the maximum allowed number of steps per episode is set to 100, providing a maximum of 100 agent-environment interactions to achieve the task. Exceeding this limit resulted in an episode reset and failure to complete the task. These failure conditions and successful task completion conditions are used to illustrate the success rate curve in Figure 6c (refer to Appendix A for more information).

In the experiments, the baseline was set at 100 ms, matching the duration of the hardware control loop frequency of the Rx200 robot (10 Hz), which is used as the default environment loop rate (10 Hz) of the Rx200 Reacher benchmark task. This selection represents the shortest action cycle time that can be used in this benchmark task because the Rx200 robot does not function properly below this duration. The training was then repeated to obtain learning curves for action cycle times of 200, 400, 600, 800, 1000, and 1400 ms. Furthermore, each run of the experiment was conducted for 30,000 steps, allowing sufficient time for each run to find the optimal policy. However, data points illustrated in Figure 6 were smoothed using a rolling mean with a window size of 10, plotted every 10th step, and shortened to the first 15K steps to improve readability.

As shown in Figure 6, increasing the action cycle time can improve performance up to a certain point compared to using the same time duration as the environment loop rate (100 ms). For this benchmark task, the learning curves for action cycle times of 600 ms and 800 ms showed the best performance, quickly stabilizing with shorter episode lengths, higher total rewards, and higher success rates. This improvement can be attributed to the balance between sufficient observation changes and the agent’s ability to interact with the environment effectively. However, as the action cycle time increased beyond 800 ms, the performance started to degrade, as the learning curves for action cycle times of 1000 ms and 1400 ms required a larger number of steps to stabilize to an optimal policy. This decline in performance is likely due to the agent receiving less frequent updates, which introduces potentially more significant errors in the policy updates, causing the agent to struggle to maintain optimal behavior.

Overall, the experiments demonstrate that while increasing the action cycle time can initially improve learning by providing more substantial observation changes, there is a threshold beyond which further increases become detrimental. Therefore, the choice of action cycle time and the use of action repeats must be balanced based on the specific requirements of the task and the capabilities of the robot. Fine-tuning these parameters is crucial for optimizing learning performance and ensuring robust real-time agent-environment interactions.

### 9.2. Impact of Environment Loop Rate on Learning

**Figure 7:**
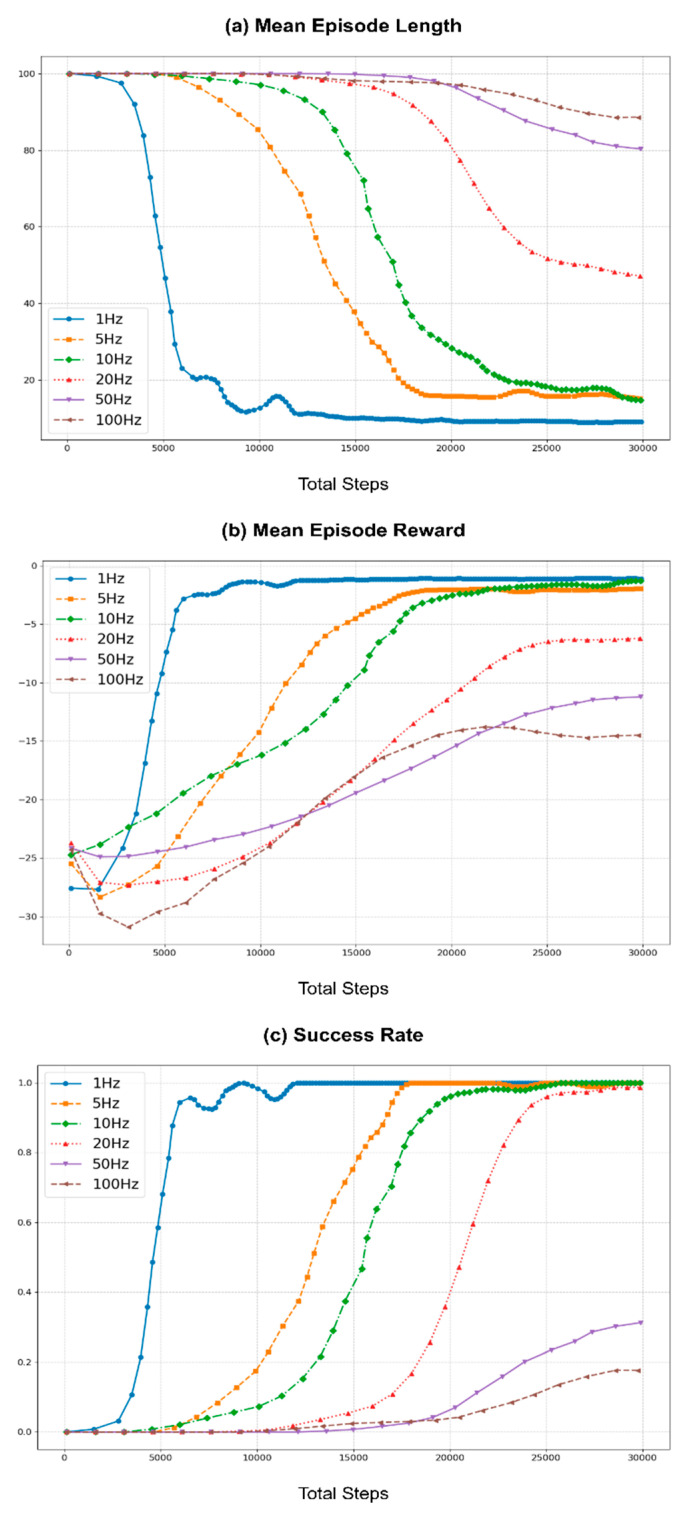
Learning performance of the Rx200 Reacher task at various environment loop rates. The results demonstrate that lower loop rates (1 and 5 Hz) than the baseline (10 Hz) improve learning performance due to larger action cycle times, which provide greater variability in observations. Higher loop rates (20, 50, and 100 Hz) degrade performance owing to instability caused by the robot’s inability to process commands and joint states effectively at frequencies above its hardware control loop frequency.

To assess the impact of various environment loop rates, the same learning process was repeated using rates of 1, 5, 10, 20, 50, and 100 Hz. Here, the action cycle time was set for each run to match the environment loop rate to simplify the task by eliminating the action repeats. Furthermore, the baseline for these experiments was set to 10 Hz to align with the hardware control loop frequency of the Rx200 robot used in the benchmark task. Similar to the previous section, Figure 7 illustrates the learning curves across different environment loop rates, with the same post-processing methods employed to enhance the readability of the curves.

As shown in Figure 7, at lower environment loop frequencies (1 and 5 Hz), the learning performance was better than the baseline of 10 Hz. This improvement can be attributed to the longer action cycle times that take larger actions (joint positions), which result in more significant variability in observations, aiding the learning process. However, the performance begins to degrade as the environment loop rate increases beyond 10 Hz. The learning curves for higher loop rates (20, 50, and 100 Hz) show increased mean episode lengths and lower mean rewards, indicating less efficient learning. This decline is due to the inability of robots to effectively process commands and read joint states at higher frequencies. Although the control commands are sent at a higher rate, the hardware control loop of the robot operates at 10 Hz, causing instability in command execution. Furthermore, as ROS controllers typically do not buffer control commands, the hardware control loop processes only the most recent command on the relevant ROS topic, leading to instability during training.

### 9.3. Empirical Evaluation of Asynchronous Scheduling of the Real-Time Environment Implementation Strategy

To empirically validate the real-time RL environment implementation strategy, the time taken for each action cycle and each actuator command cycle across numerous episodes was logged. The experiments were conducted using both the Rx200 and Ned2 Reacher, with environmental loop rates of 10 and 25 Hz, respectively. These rates correspond to a 100 ms period and 40 ms to send actuator commands to the robot, with an action cycle time set to 800 ms, providing ample time to execute action repeats. The goal was to observe the effectiveness of the asynchronous scheduling approach in managing agent-environment interactions and quantitatively measure the latency inherent in the system.

**Figure 8:**
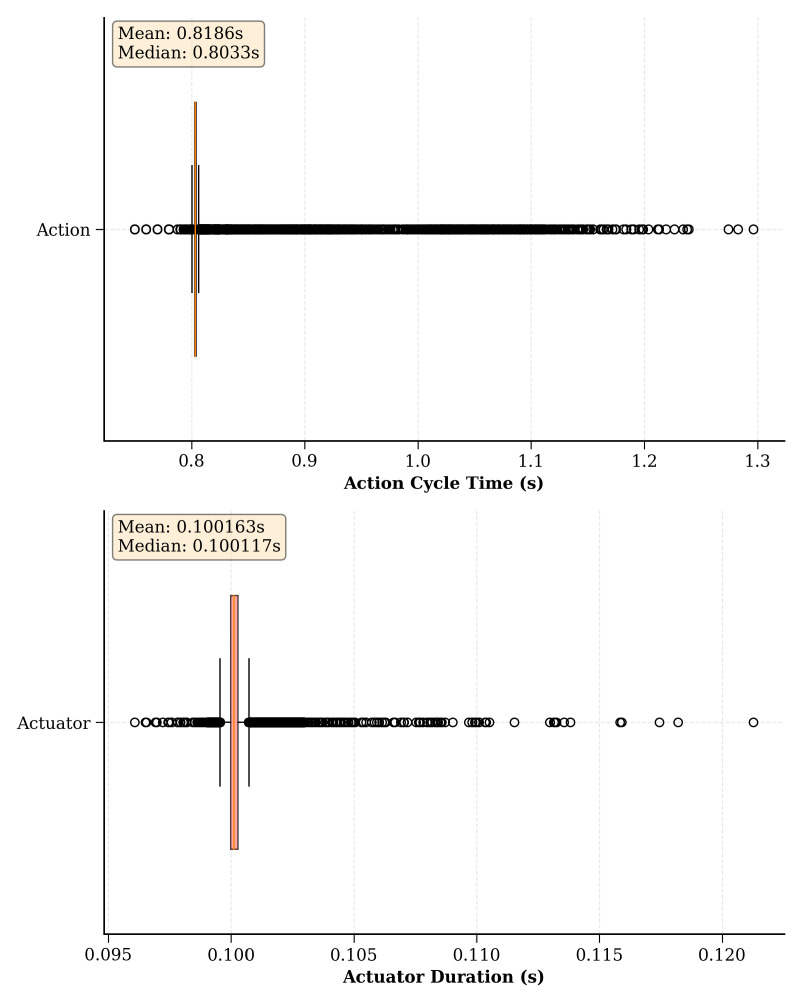
Distribution of cycle durations for the action and actuator loops in the Rx200 Reacher task using TD3 implemented with Stable-Baselines3 (SB3). While actuator durations remain consistent, the wider spread in action cycle times reflects the limitations of SB3, which is not optimized for real-time robotic training and does not support asynchronous policy updates.

The boxplot in Figure 8 depicts the distribution of cycle durations for the actuator and action cycles within the Rx200 Reacher task during training in a real-world environment. It shows a median action cycle time of 803.3 ms and a median actuator cycle of 100.11 ms, which closely approximates the respective preset threshold values for the benchmark task. Furthermore, despite the presence of some outliers, the compact interquartile ranges in both plots indicate that the system performs with a high degree of consistency, with negligible variability. However, it should be noted that the variations observed in the action cycle durations are partly due to the use of the TD3 implementation in the Stable Baselines3 (SB3) package as the learning algorithm. SB3 is a robust framework for reinforcement learning. However, it is not explicitly designed for robotic applications or real-time training scenarios and is more of a general-purpose RL library. Therefore, SB3 does not typically schedule policy updates immediately after sending an action or use asynchronous processing to update the policy, which can introduce delays and variations. Therefore, the absence of asynchronous scheduling in SB3 means that the agent waits for the policy update to be completed before proceeding with the following action. This synchronous approach can lead to slight variations in the action cycle times, as shown in the box plot.

**Figure 9:**
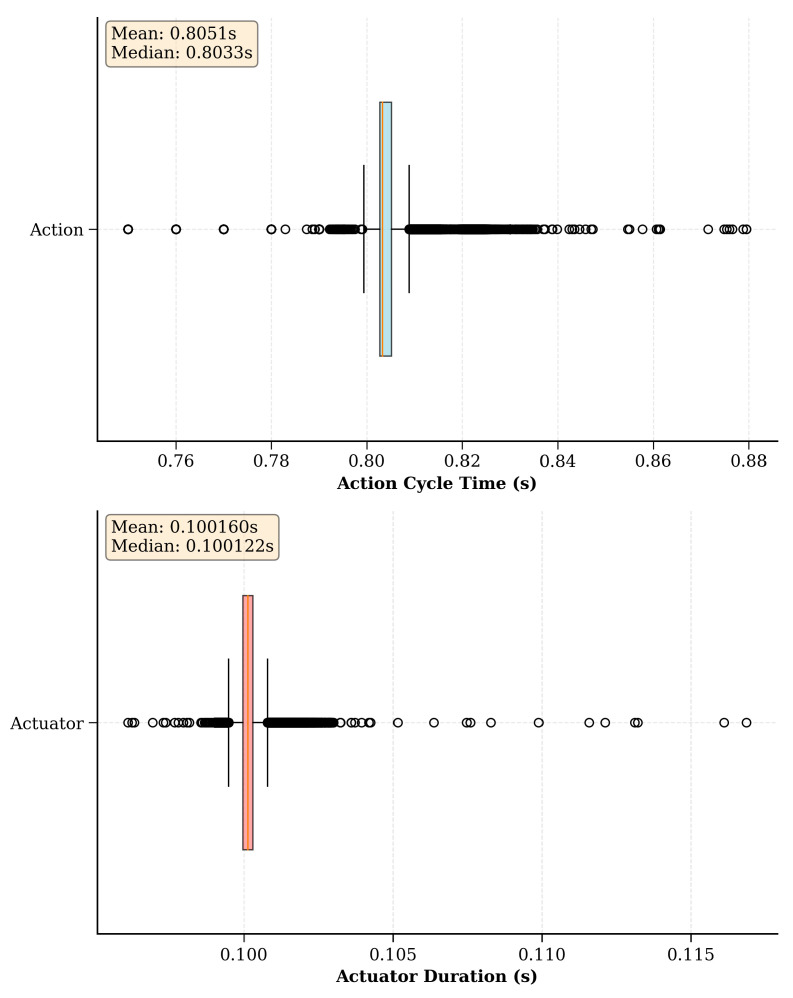
Distribution of cycle durations for the action and actuator loops in the Rx200 Reacher task using a custom TD3 implementation with asynchronous policy updates. Compared to the standard SB3-based TD3, the action cycle times exhibit a narrower interquartile range, indicating improved temporal consistency. This suggests that asynchronous scheduling of policy updates effectively reduces variability in real-time training.

One solution to this issue is to develop custom RL implementations that incorporate asynchronous policy updates [55]. This approach allows the policy to be updated in the background while the agent continues to interact with the environment, thereby reducing latency and improving the efficiency of real-time learning. By scheduling policy updates asynchronously, these methods ensure that agent-environment interactions are not interrupted, maintaining the consistency and precision required for effective real-time learning. To evaluate the impact of this approach, additional experiments were conducted using a custom TD3 implementation in which policy updates were explicitly scheduled asynchronously relative to data collection. As illustrated in Figure 9, the Rx200 Reacher demonstrated improved temporal consistency in action execution. The Rx200 Reacher task displayed a similar median action cycle time of 803.3 ms, with a narrower interquartile range than the standard SB3 implementation. This reduction in variability confirms that asynchronous scheduling effectively mitigates timing disruptions introduced by synchronous policy updates, leading to improved temporal consistency during task execution.

**Figure 10:**
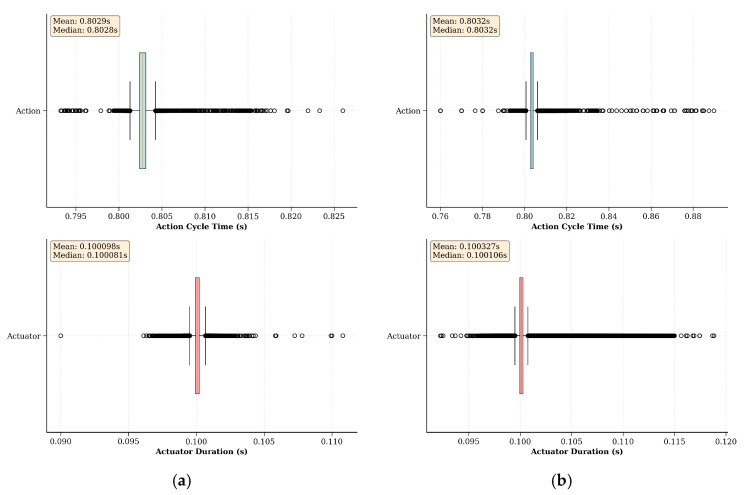

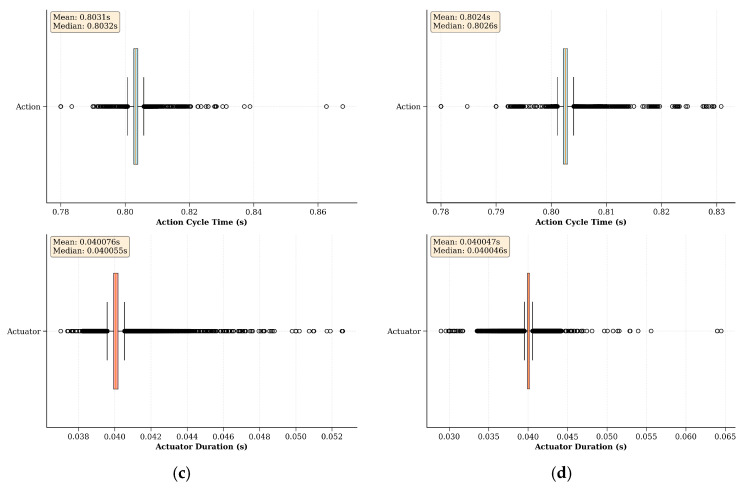
Distribution of action and actuator cycle durations during concurrent training in four environments using asynchronous policy updates. Subplots (**a**,**b**) show the results for the Rx200 Reacher task in real and simulated settings, respectively, both configured with a 100 ms actuator cycle and an 800 ms action cycle. Subplots (**c**,**d**) correspond to the Ned2 Reacher task in real and simulated environments, with a 40 ms actuator cycle and the same 800 ms action cycle. All plots exhibit narrow interquartile ranges and closely aligned medians, demonstrating consistent temporal performance and minimal variability across both platforms.

Furthermore, to evaluate the proposed approach under a high computational load, the experiments were extended to support concurrent learning across both simulated and real environments. In this setup, all four environments (two simulated and two real environments, namely, the Rx200 Reacher and Ned2 Reacher) were trained simultaneously using the asynchronous policy update mechanism. The results, as depicted in Figure 10, show that the distribution of the action and actuator cycle times across all four tasks remained consistent with those observed in the single-environment experiments. Each subplot within the composite boxplot illustrates minimal variation, with median values closely aligning with the predefined cycle thresholds, indicating that asynchronous scheduling sustains reliable timing even under multi-environment execution. These results support the proposed concurrent processing methodology, which minimizes overall system latency and facilitates real-time agent-environment interactions.

### 9.4. Discussion

All experiments in this study were conducted using a single robot in each RL environment. However, as discussed in the previous sections, the proposed UniROS framework enables the use of multiple robots in the same RL environment, particularly when they need to collaborate to complete a task. If the robots used in the task are of the same make and model, the experimenters can use the hardware control loop frequency of the robots as the environment loop rate. However, this setup could introduce additional complexities, particularly when the robots have different hardware control loop frequencies. For instance, consider that the two robots used in this study are combined in a single RL environment, where the Rx200 robot has a hardware control loop frequency of 10 Hz and the Niryo NED2 operates at 25 Hz. In such a scenario, it is desirable to use a lower hardware control loop frequency (in this scenario, 10 Hz) as the environment loop rate for the entire system to ensure synchronized operation. This approach prevents the faster robot from issuing commands more frequently than the slower robot can keep up with, thereby maintaining consistent interaction with both robots. Furthermore, pairing a slower robot like the Rx200 (10 Hz) with a more industrial-grade manipulator, such as the UR5e robot, which has a hardware control loop frequency of 125 Hz, may not be ideal. The disparity in the control loop rates can lead to inefficiencies and instability in the learning process. A slower robot can become a bottleneck, hindering the performance of a faster robot and potentially disrupting overall task execution.

Additionally, when initializing the *joint_state_controller* that publishes the robot’s joint state information, the publishing rate can be set to any frequency. This leads to the ROS controller publishing at the specified rate, even though it differs from the hardware control loop frequency of the robot. While setting a higher frequency does not impact learning, as the ROS controller publishes the same joint state information multiple times, setting a lower frequency leads to degraded performance because the agent does not receive the most up-to-date information from the robot. Therefore, the most straightforward solution is to adjust the publishing rate of the *joint_state_controller* to match the hardware control loop frequency or higher, as this ensures that each published message corresponds to an actual update from the hardware.

Similarly, suppose an external sensor in the task operates at a lower rate than the hardware control loop frequency of the robot. In such cases, it is essential to account for this in the environment loop of the RL environment. This could mean using the latest available sensor data, even if they are not updated at every loop iteration. In these scenarios, action repeats can be beneficial, especially when dealing with robots with higher hardware control loop frequencies. This makes learning easier for the RL agent by receiving observations that display more substantial changes at each instance rather than infrequent and minimal changes that make learning harder.

## 10. Use Cases

Three possible use cases of UniROS are presented, each highlighting a unique aspect of its application. The first use case demonstrates the training of a robot directly in the real world, showcasing how to utilize the framework for learning without relying on simulation. The second use case demonstrates zero-shot policy transfer from simulation to the real world, highlighting the capability of the proposed framework to transfer learned policies from simulation to the real world. Finally, the last use case demonstrates the ability of the framework to learn policies applicable to both simulated and real-world environments. In these use cases, the environment loop rate was set to 10 Hz, and the action cycle time was set to 800 ms, as this configuration showed the best results for the Rx200 Reacher task in Section 9.1. Similarly, an environment loop rate of 25 Hz and the same action cycle time of 800 ms were used for the Ned2 Reacher due to the multi-task learning setup in one of the use cases (Section 10.3), ensuring that both robots received actions at the same temporal frequency. This consistency in action dispatching across tasks facilitates stable training and improves coordination when learning shared representations in multi-robot learning setups.

### 10.1. Training Robots Directly in the Real World

**Figure 11:**
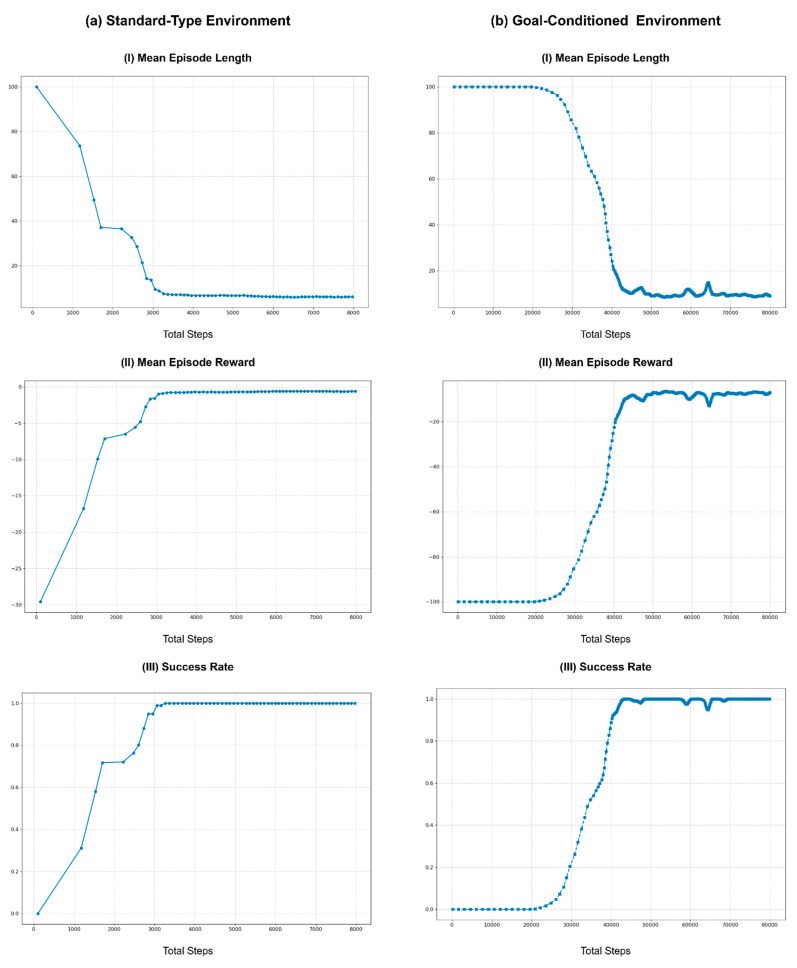
Learning curves from direct real-world training of the Rx200 robot. Part (**a**) illustrates the learning metrics for the standard-type environment using the TD3 algorithm, and Part (**b**) presents the learning metrics for the goal-conditioned environment using the TD3 + HER algorithm. These metrics collectively demonstrate the robot’s learning performance using the proposed training approach in real-world environments.

The first use case is demonstrated using the Rx200 Reacher task. Here, the physical robot was directly trained in the real world using TD3 (for a standard-type environment) and TD3 + HER (for a goal-conditioned environment). To evaluate the performance of learning, the success rate, mean total reward per episode, and number of steps (agent-environment interactions) taken by the robot to reach the goal position in an episode were plotted. Figure 11 shows the learning metrics of the trained robot in each environment. Because the experiment using the standard-type environment with the stated action cycle duration of 800 ms and environment loop rate of 10 Hz has already been conducted and showcased in Figure 6, the curves were replotted in Figure 11a for readability and to ensure that the results are easily interpretable without the complexity of multiple curves in a single figure. Therefore, to provide a clear presentation of the learning curves for both environments, Figure 11 was divided into two parts. Part (a) presents the learning metrics for the standard-type environment, and part (b) presents the learning metrics for the goal-conditioned environment. The learning curves of the next two use cases also follow the same convention.

Here, it was observed that both environments performed well on the Reacher task, as their success rates and mean rewards steadily increased, while the average steps gradually decreased as learning progressed. Furthermore, the plateau of the success rate in both environments indicates that the robot learned a near-optimal policy for the given task. These results demonstrate that the proposed study enables the direct training of robots in the real world using RL with a single experience stream.

Furthermore, it is essential to note that during the initial stage of training, challenges arose because some joints attempted to move beyond the restricted workspace. Therefore, the initial solution of simply restricting the end-effector pose to be within the workspace proved insufficient. Instead, the action (containing joint position values) was used to calculate the forward kinematics (FK) for all joints to check if any were trying to exceed the workspace limits. If any of the resulting joints were found to be out of bounds, the robot was prevented from executing the action. This additional step ensured the safety and stability of the robot during training. These findings highlight the feasibility and benefits of using the UniROS framework for direct real-world training of robotic systems, laying the groundwork for more complex future applications.

Additionally, it should be noted that the learning progress differs between standard-type and goal-conditioned environments, with the former achieving a near-optimal policy before 10K steps and the latter taking around 50K to 80K steps. A detailed explanation of this disparity is provided in Appendix C, which discusses the differences in reward architectures and environment types, highlighting how dense rewards in standard-type environments facilitate quicker convergence compared to sparse rewards in goal-conditioned environments.

### 10.2. Simulation to Real-World

**Figure 12:**
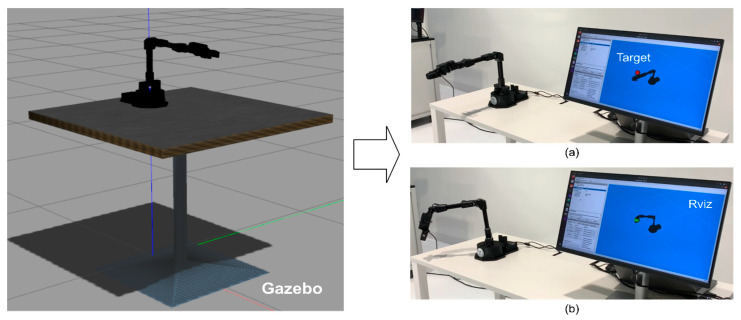
From Simulation to the Real World: The left image depicts the simulated environment in the Gazebo simulator, featuring the Rx200 robot and a table used to train the reaching task with the MultiROS package. The right image shows the real-world setup for evaluating the transferred policy, where RViz is used to visualize the randomly generated target and the status of the task. The red marker (**a**) represents the goal that the robot must reach, which turns green (**b**) upon successful completion of the task.

This section presents the experimental results obtained from training the Rx200 robot in simulation environments and subsequently transferring the learned policies to a physical robot. This experiment utilizes the simulated Rx200 Reacher task environments and employs the same RL algorithms to train the agents. These simulated environments were created following the proposed real-time implementation strategy, with the same environment loop rate and action cycle time as the previous real-world use case. Furthermore, to ensure seamless policy transfer from the simulation to the real world, the simulated environments were configured to mirror real-world conditions as closely as possible, which includes not pausing the simulation. The primary aspect of this process was to mimic the hardware control loop of the real robot within the Gazebo simulation, ensuring that the timing of the environment loop in the simulated environment closely matched that of the real-world counterpart. Figure 12 shows the constructed real-world environment in the Gazebo simulator.

Additionally, the simulated robot’s URDF file (robot description) contains the *gazebo_ros_control* (https://classic.gazebosim.org/tutorials?tut=ros_control, accessed on 17 June 2025) plugin, which loads the appropriate hardware interfaces and controller manager for the simulator to update the hardware control loop frequency of the real robot. This plugin configuration ensures that actuator commands are processed at the desired control frequency that matches the real-world RL environment. However, it was observed that manually setting this parameter in the URDF can sometimes cause the robot to exhibit unexpected behaviors in the simulation. One solution for these scenarios is to use the hardware control loop frequency of the real robot as the environment loop rate, forcing the simulated robot to receive control commands and operate at the actual hardware control loop frequency. Therefore, setting these configurations enables learning in a simulated environment that closely resembles real-world learning.

**Figure 13:**
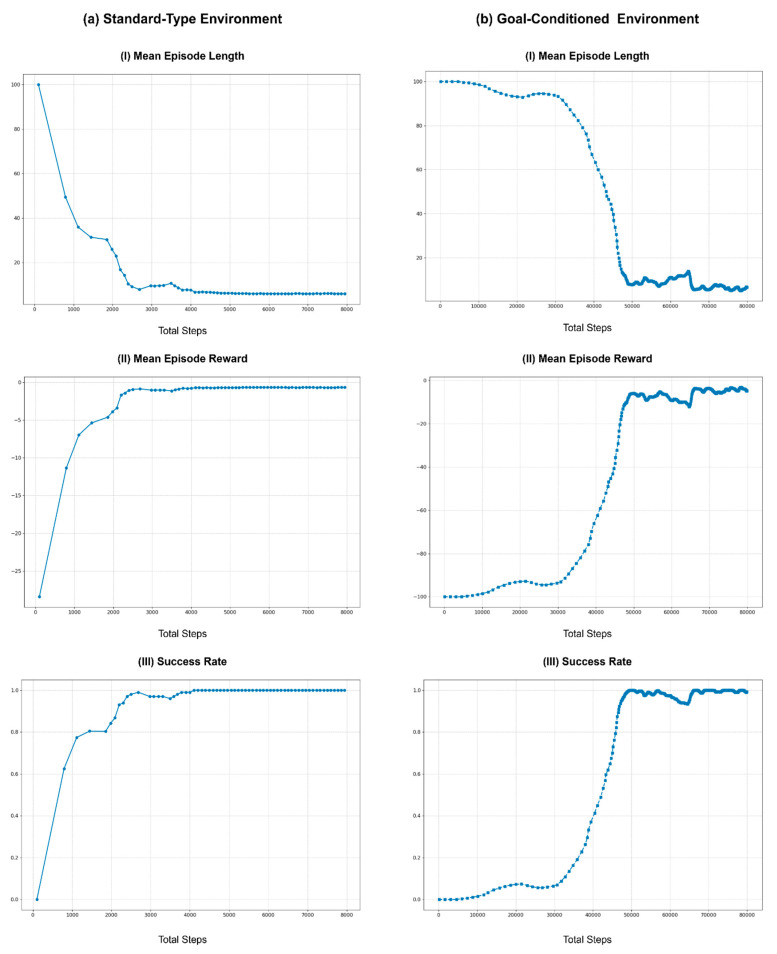
Learning metrics for training the Rx200 robot in the simulation. Part (**a**) illustrates the learning metrics for the standard-type environment using the TD3 algorithm, and Part (**b**) presents the learning metrics for the goal-conditioned environment using the TD3 + HER algorithm. These metrics demonstrate the robot’s learning performance in simulation, which was then used to transfer the learned policies to real-world environments.

As illustrated in Figure 13, the learning curves were plotted to evaluate the learning performance in both types of simulated environments. Similar to the previous use case, both agents learned nearly an optimal policy, as the success rate plateaued for the Reacher task. Furthermore, the gradual increase in the success rate and mean reward, along with the decrease in the mean episode length, indicates that the learning was stable and consistently improved throughout the training. Once the trained policies were obtained from the simulation environments, a zero-shot transfer was performed directly for the physical Rx200 robot (Figure 12) without employing any sim-to-real techniques or domain adaptation methods to bridge the reality gap. The primary objective of zero-shot transfer was to evaluate the ability of the proposed framework to generalize its learning from ROS-based simulation environments to the real world without requiring any additional training.

To evaluate policy transfer, 200 episodes of the Reach task were conducted to record the success or failure of each episode. This provides valuable insights into the performance of the transferred policies in physical robots. In this study, the trained TD3 model in the standard-type environment and the TD3 + HER model trained in the goal-conditioned environment achieved a nearly 100% success rate in the real world. This result indicates that the transferred policies can be generalized and accurately guide the physical robot without requiring additional fine-tuning.

However, it should be mentioned that while the relatively simple Rx200 Reacher task could achieve a successful zero-shot policy transfer without any additional fine-tuning, it will not necessarily be the same for all tasks. This is especially true for complex tasks involving additional sensors (cameras and lidars), as they may require domain randomization techniques, such as sampling data from multiple environments, each with different seeds and Gazebo physics parameters (which can be tuned using UniROS). In such cases, zero-shot transfer may not be feasible, and fine-tuning policies in the real world may be necessary. However, initial training in a simulated environment can also provide a good starting point for further optimization in the real world.

### 10.3. Concurrent Training in Real and Simulation Environments

This use case comprises two experiments. The first experiment demonstrates how real-world dynamics and kinematics can be learned concurrently using less expensive simulation environments to expedite the learning process. The second showcases one of the multi-robot/task learning approaches using the proposed framework and environment implementation strategy.

#### 10.3.1. Learning a Generalized Policy

This experiment aims to demonstrate the capability of the proposed framework for training a generalized policy that can perform well in both domains by leveraging knowledge from simulations and real-world data using concurrent environments. This experiment was designed using the created Rx200 Reacher real and simulated environments to learn the same Reach task. Furthermore, to be consistent with the previous use cases, one generalized policy was trained for standard-type environments (simulated and real) with dense rewards, and another for goal-conditioned environments with sparse rewards. Here, the proposed framework’s ability to execute concurrent environments was exploited to enable synchronized real-time learning in both simulation and real-world environments.

**Figure 14:**
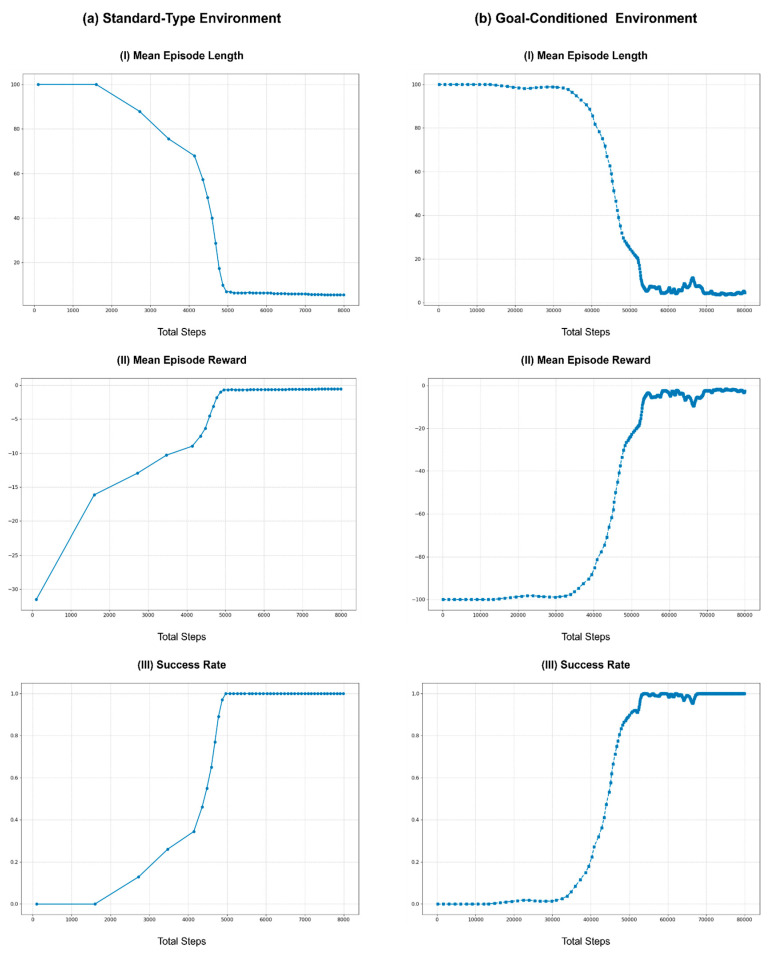
Learning metrics for simultaneously training the Rx200 robot in both real and simulated environments. Part (**a**) illustrates the learning metrics for the standard-type environment using the TD3 algorithm, and Part (**b**) presents the learning metrics for the goal-conditioned environment using the TD3 + HER algorithm. These metrics demonstrate the learning performance of the robot when trained in a combined real and simulated environment, resulting in a generalized policy applicable to both domains.

In this experiment, an iterative training approach was employed to update a single policy by sampling trajectories from both real and simulated environments, enabling the agent to integrate real-world dynamics and kinematics into the learning process. Similar to the previous use cases, a TD3-based learning strategy was applied to standard-type environments and a TD3 + HER strategy to goal-conditioned environments. The implemented learning strategy is presented in Algorithm 1. The performance of the learning process was evaluated by plotting the learning curves, as shown in Figure 14. As observed in the previous use cases, the gradual decrease in the mean episode length and increase in the rewards and success rate imply that learning was stable and consistent throughout training. Additionally, both agents learned nearly optimal policies, as indicated by the plateauing of the success rate. Furthermore, similar to the previous use case, deploying the trained agent in the respective domain yielded 100% accuracy in both types of environments.
**Algorithm 1.** Multi-task training strategy for TD3/TD3 + HER.
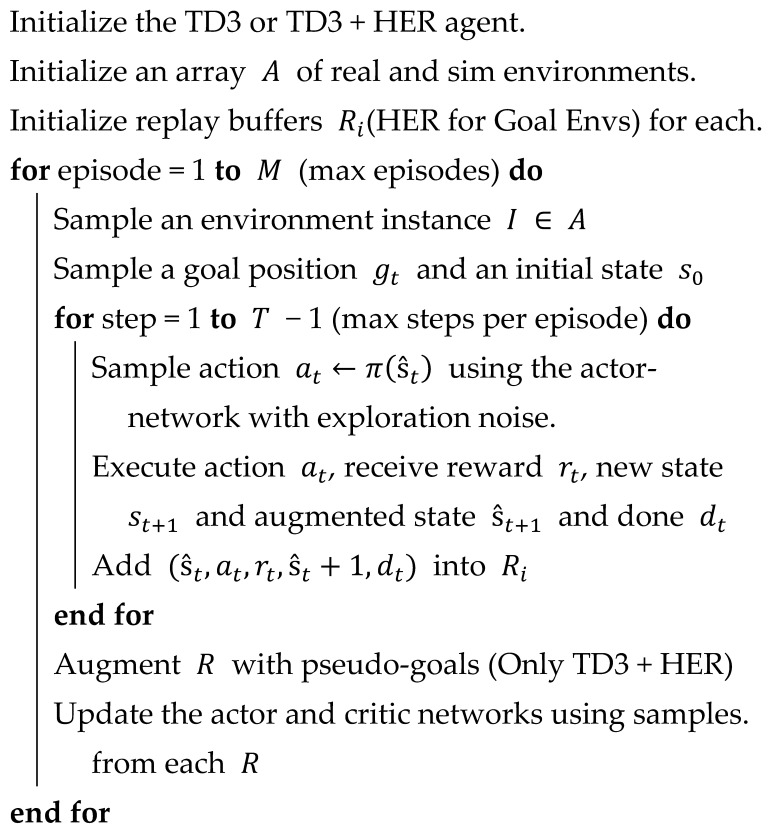


While the results illustrated in Figure 14 appear less impressive than those of the previous use cases, the main advantage of the use case lies in its demonstration of the capability to train concurrently in both real and simulated domains using the same policy. Here, the relatively simple nature of the Reach task does not fully reflect the potential benefits of this concurrent learning approach because the gap between simulation and real-world performance is minimal. However, the true strength of this concurrent learning strategy becomes more evident when it is extended to more complex robotic tasks, such as manipulation involving deformable objects, tool use, or contact-rich interactions, where discrepancies between simulated and real-world physics become more noticeable. In such scenarios, learning from real-world data is crucial for bridging the reality gap, while simulations continue to provide large-scale data for rapid iteration and policy refinement.

#### 10.3.2. Multi-Task Learning

To demonstrate one of the multi-robot/task learning approaches, the previous experiment was extended by incorporating all the created task environments of Rx200 Reacher and Ned2 Reacher into the environments array A of Algorithm 1. Then, by sampling experience from multiple environments, the agent is exposed to multiple tasks across different domains (robot types and physical/simulated instances), enabling it to learn the optimal behavior under varying conditions. Furthermore, to accommodate the differences in the observation and action spaces between the Rx200 and Ned2 Reacher environments, the agent architecture was configured using the largest observation space among the environments (from the Rx200 Reacher). For environments with smaller observation spaces, zero-padding was applied to match the input dimensions (for the Ned2 Reacher). Similarly, the largest action space across the environments (from the Ned2 Reacher) was used as the action dimension for the agent, and any additional unused actions were ignored when executing actions in environments with smaller action spaces (for the Rx200 Reacher). This design choice ensured compatibility across heterogeneous environments while maintaining the shared policy architecture.

Although more sophisticated techniques, such as using task-conditioned policies with task embeddings [56] or adaptation layers [57], could help manage heterogeneous input/output structures more elegantly, a simpler approach was deliberately chosen to keep the training pipeline minimal. This approach of padding and unifying action and observation spaces has also been used in prior multi-task reinforcement learning research [58] and serves as a reasonable baseline when dealing with a limited number of tasks and known environment interfaces.

**Figure 15:**
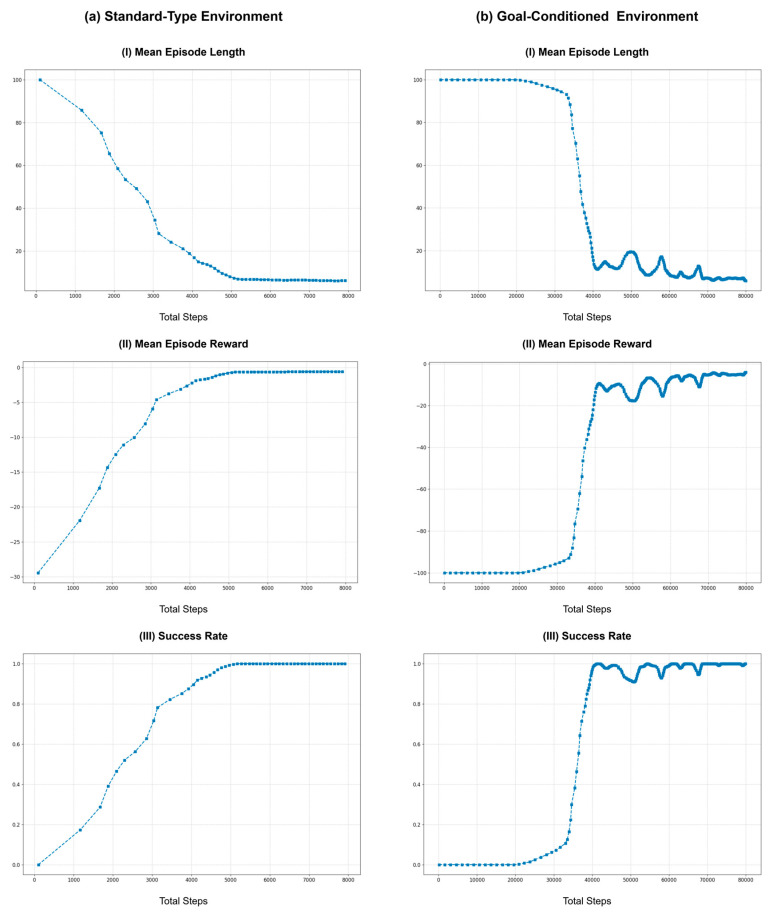
Learning metrics for multi-task learning during training across multiple environments comprising both the Rx200 and Ned2 Reacher tasks in the simulated and real domains; (**a**) shows the performance using standard dense reward environments trained with the TD3 algorithm, while (**b**) shows the performance using goal-conditioned sparse-reward environments trained with TD3 + HER. These results demonstrate stable convergence and effective policy generalization across heterogeneous robot platforms and domains.

Similar to the previous use cases, the training curves for this experiment were plotted, as shown in Figure 15. As before, it was observed that the training remained stable across both types of environments, as indicated by the gradual decrease in the mean episode length, and the agents successfully learned policies that converged toward optimal behavior with the plateauing of the success rate and the mean episode reward. The advantage of this approach lies in the agent’s ability to learn a single policy that generalizes well across multiple tasks and domain configurations, ultimately saving time and computational resources by avoiding separate training for each environment.

While the deployment of the trained agent in each environment showed similar 100% accuracy as with the previous use cases, it is important to note that these results are specific to simple Reach tasks, which have low-dimensional state and action spaces and minimal domain discrepancy. As more complex tasks are introduced, techniques like task embeddings, modular policies [59], or adaptation layers may become necessary to manage the increased diversity in task structure and dynamics. Similarly, meta-learning algorithms such as Model Agnostic Meta-learning (MAML) [60] may enable learning a model that can quickly adapt to new tasks with minimal data when the tasks are complex.

In summary, these experiments demonstrate the capability of the UniROS framework for training policies that perform well in both simulated and real-world environments. The ability to leverage both domains during training in concurrent environments without incurring synchronization bottlenecks provides a robust solution for developing versatile and adaptive robotic systems. The primary goal of these demonstrations is not to determine the best method for setting up learning tasks in the real world. Instead, it showcases the versatility and robustness of the UniROS framework, providing a platform for researchers to extend it further and adapt it to their specific research needs. Therefore, these use cases demonstrate the current capabilities of the framework and lay the groundwork for future research in more complex areas, such as robot-based multi-agent systems, multi-task learning, and meta-learning.

## 11. ROS1 vs. ROS2 Support in the UniROS Framework

While this study is primarily based on the first major version of the Robot Operating System (ROS 1), it is nearing the end of life (EOL) in 2025, as ROS 2 is emerging as the new standard. Hence, plans to upgrade the package to support ROS 2 are currently underway, starting with ROS 2 Humble (https://docs.ros.org/en/humble/index.html, accessed on 17 June 2025) and Gazebo Classic (Gazebo 11). However, it is essential not to abandon ROS1, as many robots in the ROS industrial repository and other ROS sensor packages have not yet been upgraded to ROS2, or they remain unstable. Similarly, only the latest robots are currently configured to work with ROS2, while some older generations are either discontinued, not maintained by the manufacturer, or abandoned because the companies have closed down (such as the Baxter robot). Therefore, experimenters may need to wait for the ROS community to upgrade these packages to ROS 2.

Moreover, in terms of the real-time learning strategy proposed in this study, it is worth noting that the design choices made, such as ROS timers, ROS publishers and subscribers, and action repeats, are inherently compatible with both ROS 1 and ROS 2. These components are part of the core middleware tools that remain consistent across both distributions. Consequently, the implementation strategy outlined in this work can be readily adapted to ROS2 with minimal modification, preserving its real-time characteristics and concurrent environment support.

## 12. Conclusions & Future Work

This study introduced an ROS-based unified framework designed to bridge the gap between simulated and real-world environments in robot-based reinforcement learning. The dual-package approach of the framework, which contains MultiROS for simulations and RealROS for the real world, facilitates learning across simulated and real-world scenarios by using an ROS-centric environment implementation strategy. This implementation strategy facilitates real-time decision-making by leveraging the built-in multi-threading capabilities of ROS for subscribers and timers, enabling the asynchronous and concurrent execution of operations essential for efficient RL workflows. By employing this approach and controlling robots directly through ROS’s low-level control interface, this study has effectively addressed the challenges of ROS-based real-time learning. This was demonstrated using a benchmark learning task, highlighting the low latency and dynamic interaction between the agent and environment.

Furthermore, the OpenAI Gym library has recently been deprecated and replaced by the Gymnasium (https://gymnasium.farama.org/, accessed on 17 June 2025) library [61]. Therefore, the UniROS framework has been upgraded to support Gymnasium, including the ROS-based support package for the Gymnasium-based Stable Baselines3 versions. For simplicity, only OpenAI Gym is referenced in the above sections.

Additionally, it is worth noting that all experiments focused on learning a simple Reach task, and training with multiple complex tasks that incorporate external sensors, such as vision sensors, were not explored in this study. This also includes exploring robustness strategies such as noise injection or uncertainty-aware learning to improve reliability under real-world disturbances. Therefore, one potential area for future work is to assess the framework’s capabilities for complex multi-task and meta-learning scenarios across various benchmark task environments. In particular, this would include integrating advanced simulation-to-real transfer techniques, such as domain randomization, dynamic parameter adjustment, and variability-aware training, as they are vital for bridging significant domain gaps in tasks involving more complex perception or contact dynamics. Additionally, we acknowledge that the results reported in this study are based on a single training run. Future work will include an evaluation across multiple random seeds to assess the training robustness, variance, and statistical confidence. This will help further quantify the reliability and generalization capabilities of policies trained within the UniROS framework. Furthermore, future work could investigate how different reward-shaping strategies affect learning performance, enabling better task-specific tuning.

Furthermore, it is essential to acknowledge that the robot was connected to the PC via a wired connection during the experiments, ensuring a stable and reliable communication interface. This was beneficial for conducting experiments in a controlled manner, but it may not accurately reflect the challenges that experimenters may encounter with wireless or less stable connections. Therefore, another area for future work could be to explore the implications of varying connection types on the performance and reliability of the framework. Furthermore, the robot used in this experiment is configured with effort controllers that accept *Joint Trajectory* messages. However, some robots, such as UR5e, can be configured to work with effort, position, or velocity controllers, each of which has its own advantages and disadvantages, depending on the task requirements and specific capabilities of the robot. Therefore, another area for future work could be to investigate the impact of using different types of controllers to learn the same benchmark tasks. This provides valuable insights into the effects of controller differences on learning performance.

Moreover, this study primarily discussed CPU utilization during learning and interaction with the environment. However, given that many deep RL architectures leverage GPU acceleration, future experiments should explore CPU-GPU co-utilization metrics. Understanding how GPU-based training and inference affect the timing and synchronization of agent-environment loops can further improve the real-time applicability of the framework. Similarly, future work could involve exploring other reinforcement learning algorithms and off-the-shelf RL library frameworks that are better suited for real-time control, thereby further enhancing the performance and reliability of the proposed solution.

While this study focused on manipulation tasks, the proposed framework is not limited to stationary arms. The same ROS-based abstraction can be extended to mobile robots, drones, and other robotic systems or fleets that support ROS interfaces. Therefore, future use cases could include mobile navigation, exploration, and hybrid scenarios involving both mobility and manipulations. Examining the impact of such configurations on learning efficiency, task performance, and system stability would provide valuable insights for designing robust ROS-based multi-robot RL systems.

In conclusion, the proposed ROS-based RL framework addresses the challenges of bridging simulated and real-world RL environments. Its modular design, support for concurrent environments, Python bindings for ROS, and real-time RL environment implementation strategy collectively enhance the efficiency, flexibility, reliability, and scalability of robotic reinforcement learning tasks. The experiments performed with the benchmark task further illustrate the practical applicability of the framework in real-world robotics, showcasing its potential for advancing the field of reinforcement learning. Therefore, we encourage the community to build upon this foundation by exploring more intricate tasks and environments and pushing the boundaries of what is achievable in robotic reinforcement learning.

## Figures and Tables

**Table 1:** Comparison of UniROS with related reinforcement learning frameworks for robot control.

Frameworks	UniROS	OpenAI_ROS	gym-gazebo2	FRobs_RL	ros-gazebo-gym	SenseAct	Orbit
Real-Time Capability	✓	✗	Limited	✗	✗	✓	✓
Supports Real Robots	✓	✓	✓	✓	✓	✓	Partial
Simulation Support	✓	✓	✓	✓	✓	✗	✓
Sim-to-Real Support	✓	Partial	Partial	Partial	Partial	✗	✓
ROS Integration	✓	✓	✓	✓	✓	✗	✓
Concurrent Multi-Robot Support	✓	✗	✗	✗	✗	✗	Partial
Latency Handling/Modeling	✓	✗	✗	✗	✗	✓	Partial
Python API for Env Management	✓	✗	Manual	✗	Partial	✗	✓
Actively Maintained	✓	✗	✗	✓	✓	✗	✓
Open Source	✓	✓	✓	✓	✓	✓	Partial

**Table 2:** Functional overview of BaseEnv, RobotEnv, and TaskEnv classes within the RealROS.

Env	Main Responsibility	Interacts With	Key Methods
BaseEnv	Core RL environment loop interface	TaskEnv, RobotEnv, RL agent	step(), reset(), close(),
RobotEnv	Robot-specific sensor/actuator API	ROS drivers, TaskEnv	_check_connection_and_readiness()
TaskEnv	Task-specific logic (reward, done)	TaskEnv, BaseEnv	_set_action(), _get_observation(), _get_reward(), _compute_terminated(), _set_init_params()

## Data Availability

The source code for the UniROS framework, along with all associated simulation environments, experimental scripts, and configuration files used in this study, is publicly available on GitHub at https://github.com/sri-tus-ie/UniROS (accessed on 17 June 2025) and https://github.com/ncbdrck/uniros_support_materials (accessed on 17 June 2025).

## Abbreviations

The following abbreviations are used in this manuscript:

ROS	Robot Operating System
RL	Reinforcement Learning
MDP	Markov Decision Process
CLI	Command Line Interface
API	Application Programming Interface
DRL	Deep Reinforcement Learning
DQN	Deep Q-Network
TD3	Twin Delayed Deep Deterministic Policy Gradient
SB3	Stable Baselines3
GIL	Global Interpreter Lock
URDF	Universal Robot Description Format
IK	Inverse Kinematics
FK	Forward Kinematics
EE	End-Effector
DOF	Degree of Freedom
HER	Hindsight Experience Replay
SSH	Secure Shell
PC	Personal Computer
VRAM	Video Random Access Memory
GPU	Graphics Processing Unit
CPU	Central Processing Unit

## Appendix A

In this study, four RL environments were designed for each robot using the UniROS framework. Two real-world environments were created using the proposed RealROS package, and two simulation environments were created using the MultiROS package. Table A1 and Table A2 present an overview of the environments of the respective robots.

**Table A1:** Overview of the Rx200 Reacher environments.

	Standard Env	Goal Env	
Action Space	Joint positions		
	5 actions (Continuous)		
Observation	EE position	3	
	Cartesian Displacement	3	
	Euclidean distance (EE to Reach goal)	1	
	Current Joint values	8	
	Previous action	5	
Achieved Goal	N/A	EE position	3
Desired Goal	N/A	Goal position	3
Reward Architecture	Dense	Dense	
	Sparse	Sparse	

**Table A2:** Overview of the Ned2 Reacher environments.

	Standard Env	Goal Env	
Action Space	Joint positions		
	6 actions (Continuous)		
Observation	EE position	3	
	Cartesian Displacement	3	
	Euclidean distance (EE to Reach goal)	1	
	Current Joint values	6	
	Previous action	6	
Achieved Goal	N/A	EE position	3
Desired Goal	N/A	Goal position	3
Reward Architecture	Dense	Dense	
	Sparse	Sparse	

In the environments, the 3D position of the end-effector $\left(x_{a},\;y_{a},\;z_{a}\right)$ and the current goal $\left(x_{g},\;y_{g},\;z_{g}\right)$ are used to determine the relative Cartesian displacement. This is determined using the following equations:

Displacement EE to Goal =Current Goal −Current EE Position

The Euclidean distance (*d_e_*) from the current position of the end-effector to the current goal position is calculated as follows:

$$Sparse\;Reward=\{\begin{array}{cc} +1 & \mathrm{if}\;d_{e}\leq \varepsilon_{r}\;(\mathrm{reach}\;\mathrm{tolerance})\\ -1 & \mathrm{otherwise} \end{array}$$

Since this study is based on ROS-based environments, the dense reward architecture differs from the others, which only use the reward as a negative value of the Euclidean distance between the end-effector and goal positions. The dense reward architecture of the Reacher task is depicted in Algorithm A1.

**Algorithm A1.** Dense reward architecture.

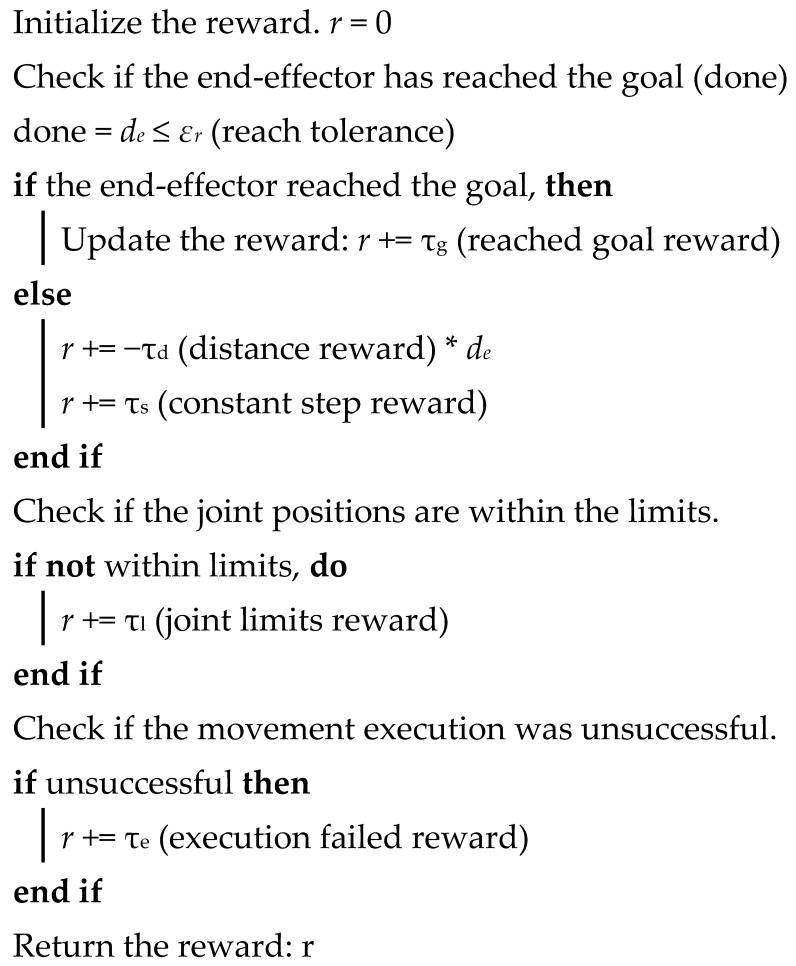

Here, in the above dense reward architecture, the reached goal reward τ_g_ is set to 20.0, the multiplier distance reward τ_d_ to 2.0, the constant step reward τ_s_ to −0.5, the not within joint limits reward τ_l_ to −2.0, and execution failed reward τ_e_ to −5.0. Furthermore, this Reach task is episodic and terminates when *d_e_ ≤ ε_r_* or the agent exceeds the maximum allowed step in the environment. Additionally, to obtain the success rate graphs, the environment set info[‘is_success’] = True when the task is completed successfully and info[‘is_success’] = False otherwise.

## Appendix B

This section presents the hyperparameters used in experiments to evaluate the real-time environment implementation strategy and three use cases of the proposed framework. All standard-type environments with dense rewards were trained using the TD3 algorithm, and goal-conditioned environments with sparse rewards were trained using the TD3 + HER algorithm. Furthermore, to be consistent, the same hyperparameters in Table A3 were used for all TD3, and those in Table A4 for TD3 + HER implementation.

**Table A3:** Hyperparameters for TD3 implementation.

Actor and Critic learning rate	0.0003
Size of the replay buffer	1,000,000
Minibatch size for each gradient update	256
Soft update coefficient	0.005
Discount factor	0.99
Entropy regularization coefficient	0.01
Maximum episode time steps	100
Success distance threshold	0.02 m
hidden layers	400, 300
Optimizer	Adam

**Table A4:** Hyperparameters for TD3 + HER implementation.

Actor and Critic learning rate	0.0003
Size of the HER replay buffer	1,000,000
Goal selection strategy	future
Number of future goals	4
Minibatch size for each gradient update	256
soft update coefficient	0.005
Discount factor	0.99
Entropy regularization coefficient	0.01
Maximum episode time steps	100
Success distance threshold	0.02 m
Hidden layers	400, 300
Optimizer	Adam
Policy delay	2
Target policy noise (smoothing noise)	0.2
Limit for the absolute value of target policy smoothing noise	0.5

## Appendix C

In all three use cases in Section 10, the learning was stable, and each achieved a near-optimal policy within 10K steps for standard-type environments and 60K for goal-conditioned environments. This is primarily due to the efficacy of the proposed RL environment implementation strategy and stability of the TD3 algorithm. It should be noted that the distinction in learning progress between standard-type and goal-conditioned environments does not indicate that one is superior to the other. The main reason for this disparity is the selected reward structure of each environment type, as it dictates the pace of learning. Therefore, the faster learning time (fewer steps) for the standard-type environments can be attributed to the dense rewards providing more frequent and informative feedback to the agent, which helps with quicker convergence and policy optimization compared to goal-conditioned environments with sparse rewards. The main benefit of using sparse rewards in robotics is that they encourage the development of more robust and generalizable policies due to the agent exploring more to achieve the ultimate task goal rather than optimizing for intermediate rewards [62]. This makes learning behaviors more adaptable to real-world scenarios. Moreover, carefully hand-coding a dense reward architecture may not be feasible when the task is complex, as in many robot applications.
